# Mass Spectrometry for Investigation of Natural Dyes in Historical Textiles: Unveiling the Mystery behind Safflower-Dyed Fibers

**DOI:** 10.1021/jasms.1c00195

**Published:** 2021-09-03

**Authors:** Katarzyna Lech, Jakub Nawała, Stanisław Popiel

**Affiliations:** † Faculty of Chemistry, Warsaw University of Technology, Noakowskiego 3, 00-664 Warsaw, Poland; ‡ Military University of Technology, Institute of Chemistry, Gen. S. Kaliskiego 2, 00-908 Warsaw, Poland

**Keywords:** high-performance liquid chromatography, tandem mass spectrometry, safflower, quinochalcone *C*-glycosides, tri-*p*-coumaroylspermidine, historical textiles

## Abstract

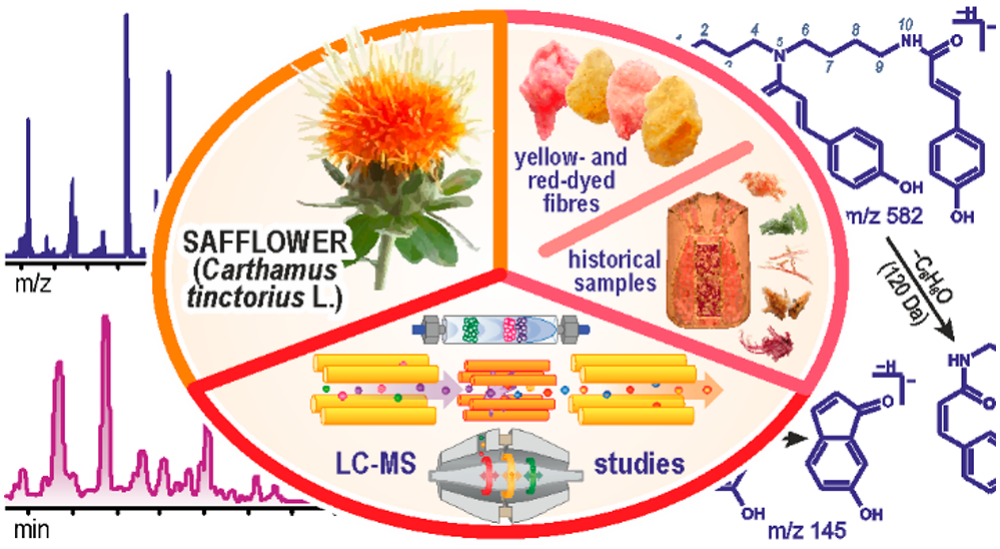

Safflower (*Carthamus tinctorius* L.) petals, depending on the nature of a dyeing bath, dye fibers yellow or red. This is due to the presence of two kinds of components, water-soluble yellow colorants and alkali-soluble red compounds. In this study, safflower-yellow- and safflower-red-dyed silk, cotton, and wool fibers were investigated using high- or ultrahigh-performance liquid chromatography hyphenated with spectrophotometry and tandem mass spectrometry (HPLC–UV–vis–ESI-MS/MS) and high-resolution Orbitrap mass spectrometry (HPLC–HESI-HRMS) in order to identify the natural dye in historical textiles. This way, several quinochalcone *C*-glycosides were separated and characterized. Their low- and high-resolution MS/MS spectra expanded the database of natural colorants in cultural heritage objects. Moreover, the colorless ct-markers (with a hitherto unknown structure) present in all safflower-dyed fabrics, regardless of the color or preservation conditions, were revealed to be *E*/*Z* stereoisomers of *N*^1^*,N*^5^*,N*^10^-tri-*p*-coumaroylspermidine. Since most of the standards was not available, discussion on possible molecular structures was provided. As a consequence, the analytical investigation of the reference fibers dyed with safflower demonstrated that the dye composition varies, depending on the dyeing conditions and type of fiber. Moreover, it was proven that carthamin, although alkali soluble, can be successfully released with a mild extraction method, without its hydrolysis under these conditions. The results helped us to characterize threads sampled from 16th to 18thcentury textiles of European and Near Eastern origin. It has completed the picture of natural dyes used in the most valuable textiles availed in liturgical vestments from the collections of Krakow churches.

## Introduction

Safflower (*Carthamus tinctorius* L.), a native of the arid areas having seasonal rains from Egypt to India, has been known and cultivated since antiquity.^1−3^ It was used for dyeing red in China as early as the Zhou (1046–256 BC) dynasty and the Han (202 BC-220 AD) dynasty,^4^ in Japan at least in the second century BC, and in Persia in the sixth century BC.^1^ However, since the safflower red colors (sometimes also pinks), although bright and beautiful, were also very transitory, initial baths of yellow dyes were used prior to the essential dyeing stage.^2,4^ Thus, safflower was used in combination with Amur cork tree, turmeric, or smoketree to increase both the fastness and the intensity of the colors in many Chinese textiles^4^ as well as together with gardenia yellow to obtain the distinctive red of the rising sun on the Japanese flag. Although in Egypt the oldest certain linen cloth dyed pink with safflower are dated back to the 21st Dynasty (1069–945 BC), it is known that *Carthamus* has been grown there since the 18th Dynasty (1550–1292 BC).^2^ Although this dye has never been overly popular in modern Europe, until recently in Great Britain a cotton “red tape”, that tied government papers, was dyed with safflower.^2,5^ Moreover, the dye was also converted into a pigment for various purposes, including cosmetics, as a gilding substance and an artists’ pigment for painting.^6^

The beautiful deep safflower reds were, however, very expensive, since it needed several, even 20, successive dyeing baths to get such intense colors.^2^ On the other hand, the ability of safflower to dye yellow had not been fully exploited in ancient times. Only one document of the Jin (265–420) dynasty has hitherto mentioned the use of safflower for dyeing yellow in China at that time.^4^ Nevertheless, it was sometimes recommended as an initial or ground bath for dyeing scarlet red. Moreover, safflower was used in India to dye turbans yellow or green (together with indigo). It was also found in Persian textiles dated back to the eighth or ninth century and used in precious chasubles.^2^

Thus, safflower was used on silk, cotton, and linen for dyeing red and on silk and wool to obtain yellow colors.^1,2,4,5^ This was possible due to the different properties of its color compounds classified into *C*-glucosyl quinochalcones. Carthamin, an alkali-soluble red compound synthesized at the late blooming phase, is essential for dyeing pink, scarlet, carmine, and deep red.^2^ However, apart from carthamin, the petals also contain large amounts of water-soluble yellow compounds, such as anhydrosafflor yellow B, safflor yellow A, hydroxysafflor yellow A, hydroxysafflor yellow B, hydroxysafflor yellow C, safflomin A, safflomin C, tinctormin, or cartormin (Supporting Information, Figure S1). Their presence in a water bath determines the ability to dye yellow or orange (very often on alum mordant), effectively preventing red color. However, dyeing red is possible as long as the water-soluble yellow compounds are formerly removed from the petals.^1,2^

Despite the wide variety of quinochalcone *C*-glucosides, safflower has so far been identified in historical fabrics mostly due to the presence of four other colorless isomers called ct-components, which have been present in the extracts of both yellow and red threads, no matter how severe the degradation or hydrolysis of other markers has gone.^7−9^ Thus, although the structures of the ct-components had hitherto been unknown to researchers of historical textiles, they have been used as the absolute markers of safflower.^10^ Unfortunately, although the presence of these compounds undoubtedly demonstrates the use of the dye, the ct-components, unlike quinochalcone *C*-glucosides, do not provide any information about the original safflower-red or safflower-yellow color of fabrics.

Since safflower, like other natural dyes, contains several color compounds, their prior separation is extremely useful for correct and complete identification of the dye. It is achieved with high-performance liquid chromatography (HPLC) or ultraperformance liquid chromatography (UPLC), that together with spectrophotometric detection (UV–vis) and/or electrospray mass spectrometric detection (ESI-MS) is the most widely adopted technique in the research of natural organic dyes in heritage objects.^1,11,12^ MS offers the combination of high sensitivity and selectivity, wide applicability to various dye classes, and ability to their quantification. However, despite the many advantages of the HPLC–MS combination and the high selectivity of the technique over other systems, it also has some weaknesses deriving from the essence of the ESI interface. The competition for ionization efficiency in the ion source, between the analytes of interest and other endogenous or exogenous matrix compounds (salts, proteins, phospholipids, polymers, plasticizer, surfactants, etc.), can cause the ion suppression phenomenon.^13,14^ It may affect the sensitivity, precision, and accuracy of the assay and, thus, both the quantitative and qualitative analysis.^15−18^ Thus, besides proper sample preparation (and also its cleanup,^14−17,19^ if necessary), it is also important to optimize the ionization (as well as fragmentation) parameters to reduce background signals, minimize the formation of adducts, and maximize the signal-to-noise ratio improving the sensitivity of the MS detector with regard to analytes. Moreover, tandem mass spectrometry (MS/MS) or high-resolution mass spectrometry (HRMS) also provides structural information, thereby enabling the identification of analytes.^20−23^ This is particularly important and advantageous for new dye markers.^24−31^

Classic ESI-MS/MS fragmentation mechanisms (comprising remote hydrogen rearrangements, retro-Diels–Alder (RDA) reactions, retro-ene reactions, retro-heteroene reactions, displacement reactions, charge remote fragmentations, inductive cleavage, simple α-elimination, aromatic eliminations, and radical eliminations^21^) resulting in neutral losses^20,32−34^ play a key role in the structure elucidation of small natural compounds. Although the occurrence of these reactions is related to the stabilization of a product ion by resonance, different analyzers and different collision gases can cause differences in the fragmentation behaviors of the same molecule. The fragmentation pathways and/or the MS/MS ion intensities depend on the competition of kinetic and thermodynamic factors.^21^ However, collision-induced dissociation (CID) and higher-energy collisional dissociation (HCD) are complementary tools for the characterization of unknown compounds.

Safflower has been identified by chromatographic analysis on shrouds and wrappings of Egyptian mummies (the 25th and 26th dynasties, 700–500 BCE),^35^ Chinese silks and silk embroideries (the 7th–10th and 12th–18th centuries),^10,36,37^ Persian (Safavid) and Indian (Mughal) velvets (the 16th–18th centuries),^38^ Italian silk (the 16th century),^10^ silk tapestries of Florentine manufacture (the 16th century),^39^ an inner lining of Italian doublet (the 17th century), and Russian pocket (the 19th century)^9^ varying in color from beige through orange and pink to red.

This work provides a detailed and comprehensive study on safflower-dyed fibers (silk, cotton, and wool) by the use of HPLC-UV–vis–ESI-MS/MS and UPLC–HESI-HRMS/MS. It led to the structure elucidation of six colorless markers (formerly known as ct compounds), as well as to identification of other compounds (ten quinochalcones and nine other compounds) extracted from dyed fibers. In consequence, the high- and low-resolution MS/MS spectra were used to update the database of markers for the identification of natural dyes in historical and archeological objects. The results enabled through characterization of orange, red, and green threads sampled from 16th- to 18th-century silk textiles of European and Near Eastern origin.

## Experimental Section

### Apparatus

Separation and identification of the colorants was carried out using a 1220 Infinity II LC System (Agilent Technologies, USA) with a Zorbax SB-Phenyl column (4.6 × 150 mm, 3.5 μm, 80 Å, Agilent Technologies), a Zorbax SB-Phenyl precolumn (4.6 × 12.5 mm, 5.0 μm, Agilent Technologies), and a mobile phase composed of (A) 0.1% formic acid in water (v/v) and (B) 0.1% formic acid in methanol (v/v) and delivered with a total flow rate of 0.5 mL·min^–1^. The LC system was combined in series with two variable wavelength detectors, a 1200 Series one and a 1220 Series one (Agilent Technologies, Germany), as well as with a 6460 Triple Quad mass spectrometer with JetStream Technology (Agilent Technologies, USA). A full survey scan MS was acquired in negative-ion mode from *m*/*z* 100–1000 with an orifice voltage (fragmentor) 135 V. The product ion spectra (MS/MS) were acquired using various collision energies (CE), between 15 and 45 V, from *m*/*z* 50 to the *m*/*z* value of the parent ion +20 to achieve an upper limit around 20 *m*/*z* above the *m*/*z* of each fragmented ion. Historical samples were analyzed in both negative and positive dynamic multiple reaction monitoring (dMRM) modes using the HPLC–UV–vis–ESI-MS method (all LC–MS details are presented in refs (29 and 30)) extended with the safflower markers (the retention times, selected transitions, fragmentor values, and CEs for safflower markers are presented in Table 1). The analyses were controlled by a MassHunter Workstation software (Agilent Technologies, USA).

**Table 1:** Color Compounds Present in Safflower-Dyed Fibers Revealed by HPLC–UV–vis–ESI-MS/MS^a^

						red-dyed		yellow-dyed	
no.	compd name	*t*_R_, min	[M – H]^−^, *m/z*	frag, V	product ions, *m/z* (CE, V)	silk	cotton	silk	wool
1	hydroxysafflor yellow A	6.5	611	170	491 (20), 403 (25), 325 (30)	–	–	√√	√√
2	*p*-hydroxybezoic acid	7.4	137	90	93 (12)	√	√	√	√
3	hydroxysafflor yellow B/C	9.0	611	170	521 (25), 313 (45)	–	–	√√	√√
4	*p*-hydroxystyrene	9.1	121	90	77 (8)	√√	√	√√	√√
5	anhydrosafflor yellow B isomer	10.0	1043	130	1025 (35), 923 (45), 449 (50)	–	–	√	–
6	*p*-coumaric acid	10.5	163	90	119 (12)	–	–	–	√
7	quercetin *O-*hexoside	10.8	463	130	301 (20), 300 (20), 271 (35), 243 (35)	–	–	√√	√√
8	anhydrosafflor yellow B	11.7	1043	130	1025 (35), 923 (35), 449 (45)	–	–	√	√
9	rutin	12.1	609	210	301 (30), 300 (38), 271 (45)	–	–	√	√
10	hyperoside	12.7	463	170	300 (22), 271 (45)	–	–	√√	√
11	deglucosyl hydroxysafflor yellow A	13.3	449	150	299 (20), 286 (20), 207 (20), 119 (35)	√√	√√	√	√
12	unknown compound (ctX)	13.5	476	130	356 (15), 268 (23), 168 (35), 119 (45)	√√	√√	–	–
13	safflor yellow A	13.7	593	130	447 (15), 327 (25), 297 (30), 119 (38)	–	–	√√	√
14	cartormin	13.9	574	130	454 (22), 424 (28), 411 (35), 364 (45)	√	√	√	√
15	kaempferol *O*-deoxyhexosyl-hexoside	14.9	593	170	285 (35)	–	–	√	√√
16	safflomin C	16.0	613	170	551 (20), 361 (35), 287 (30)	–	–	√	√
17	quercetin	18.2	301	130	179 (13), 151 (13), 107 (25)	√	–	√√	√
18	tri*p*-coumaroylspermidine (ct1)	21.4	582	170	462 (25), 342 (35), 119 (45)	√√	√	√√	√√
19	tri*p*-coumaroylspermidine (ct2)	22.3	582	170	462 (25), 342 (35), 119 (45)	√√	√	√√	√√
20	tri*p*-coumaroylspermidine (ct3)	22.5							
21	tri*p*-coumaroylspermidine (ct4)	23.1	582	170	462 (25), 342 (35), 119 (45)	√√	√	√√	√√
22	tri*p*-coumaroylspermidine (ct5)	23.3							
23	tri*p*-coumaroylspermidine (ct6)	24.1	582	170	462 (25), 342 (35), 119 (45)	√√	√	√√	√
24	apigenin	24.3	269	130	117 (27)	√	√	√	√
25	carthamin	29.5	909	170	501 (25), 407 (25), 287 (35)	√	√√	–	–
26	unknown compound (ctY)	12.9	477	130	459 (22), 289 (25), 119 (45)	N/A	N/A	N/A	N/A
27	unknown compound (cyZ)	17.4	562	130	442 (12), 416 (15)	N/A	N/A	N/A	N/A

a √√, dominant compound; √, minor compound; −, not present.

High-accuracy and high-resolution MS analyses of safflower markers were carried out using a Q Exactive Plus mass spectrometer (Thermo Fisher Scientific, Bremen, Germany) and a Thermo Scientific Vanquish system with an autosampler, a column thermostat, and a binary pump (Thermo Fisher Scientific, Bremen, Germany). The UPLC system was equipped with a reversed-phase analytical column, Hypersil GOLD aQ C18 (150 × 2.1 mm, 1.9 μm, Thermo Scientific), with the use of a mobile phase composed of the same A and B solvents and the same total flow rate as described above for the HPLC–UV–vis–ESI-MS method. The following gradient program was applied: 0 min, 10% solvent B; 15 min, 60% solvent B; 20 min, 70% solvent B; 27 min, 100% solvent B; 35 min, 100% solvent B. Detection was operated with a heated electrospray ionization (HESI) interface in negative ionization mode. The following parameters of HESI source were applied: sheath gas flow 55 L·min^–1^, aux gas flow 15 Arb, sweep gas flow rate 5 Arb, spray voltage 2.5 kV, spray current 5.5 μA, capillary temperature 270 °C, aux gas heater temperature 450 °C. A full survey scan MS (from *m*/*z* 100–1000) was acquired in the Orbitrap with a resolution of 140,000. The automatic gain control (AGC) target for MS1 was set as 5 × 10^6^ and ion filling time set as 200 ms. Selected ions were isolated in a 3 s cycle and fragmented using high-energy collisional dissociation (HCD) with collision energy from 10 to 40 V and detected at a mass resolution of 70,000 at *m*/*z* 200. The AGC target for MS/MS was set at 5 × 10^6^ and ion-filling time set at 200 ms. Dynamic exclusion was set for 30 s with a 10-ppm mass window.

Extraction of the colorants from fibers was performed with a Branson 1210 ultrasonic bath (Danbury, USA) and a Memmert WB 10 water bath (Schwabach, Germany). The extract was separated from the residue using an MPW Med. Instruments MPW-350R centrifuge (Warsaw, Poland).

### Chemicals and Materials

Apigenin and *p*-coumaric acid were purchased from Fluka (Buchs, Switzerland); *p*-hydroxybenzoic acid from Sigma-Aldrich (St. Louis, MO); hyperoside and rutin from ChromaDex (Santa Ana, CA); and quercetin from Riedel-de Haën (Seelze, Germany). Safflower (*Carthamus tinctorius* L.) petals were purchased from Kremer-Pigmente (Aichstetten, Germany), methanol (LC/MS purity) from POCH (Gliwice, Poland), formic acid (LC/MS purity) from Fisher Scientific (Fair Lawn, NJ), and hydrochloric acid (analytical grade, 35–38%)from AppliChem (Darmstadt, Germany). Demineralized water was made using a Milli-Q system Model Millipore Elix 3 (France). Raw silk and cotton fibers were received from silkworm cocoons and cotton balls that were purchased at a local market (Warsaw, Poland). Sheep wool came from a rural farm in the Kuczbork commune of Northern Mazovia (Poland).

The historical thread samples originated from 16th to 18th century textiles used in the vestments owned by Krakow churches (all the samples with detailed description are listed in Table 3).

### Sample Preparation

Safflower petals bundled up in a mesh pouch were immersed in cold water and left to soak for 1 h, squeezing periodically. After this time the pouch with petals was taken out from the yellow solution and squished several times to drain from it the rest of the solvent with coloring components; the received solution (dyeing bath 1) was used to dye yellow later on. Water baths were changed seven times, until yellow colorants were washed away. Finally, the pouch was left in water to soak overnight. The next day the pouch was squished and then immersed in an ammonia–water solution (pH 10.5) for 1 h to obtain the peculiar orange-brown extract. After that petals were removed, and the pH of the solution was reduced to 6.0 using citric acid (the extract became pinkish red); the received solution (dyeing bath 2) was used to dye red.

To dye yellow, silk and wool fibers were immersed in a yellow dyeing bath (1). The solution was heated up to 50 °C (not more than 60 °C) and the fibers were soaked at this temperature for 45 min. After that they were washed with water and allowed to dry in air. To dye red, silk and cotton fibers were immersed in an orange-brown dyeing bath (2) at room temperature and left there overnight. After the fibers became pinkish, they were washed with water and left to dry in a dark place. The ratio of fibers to dye to water was about 4:1:300 (m/m/v).

Safflower-dyed fibers and historical samples were extracted using the procedure proposed by Lech,^29^ mostly with the water–methanol–formic acid method (50 μL of the mixture (8:9:3, v/v/v); 20 min of ultrasounds, then 25 min in 60 °C; dilution with 50 μL of a methanol–water mixture (9:8, v/v)); only two green historical fibers were also extracted with the DMSO method (50 μL of DMSO; 10 min of ultrasounds, then 20 min in 60 °C; dilution with 50 μL of methanol).

## Results and Discussion

**Figure 1:**
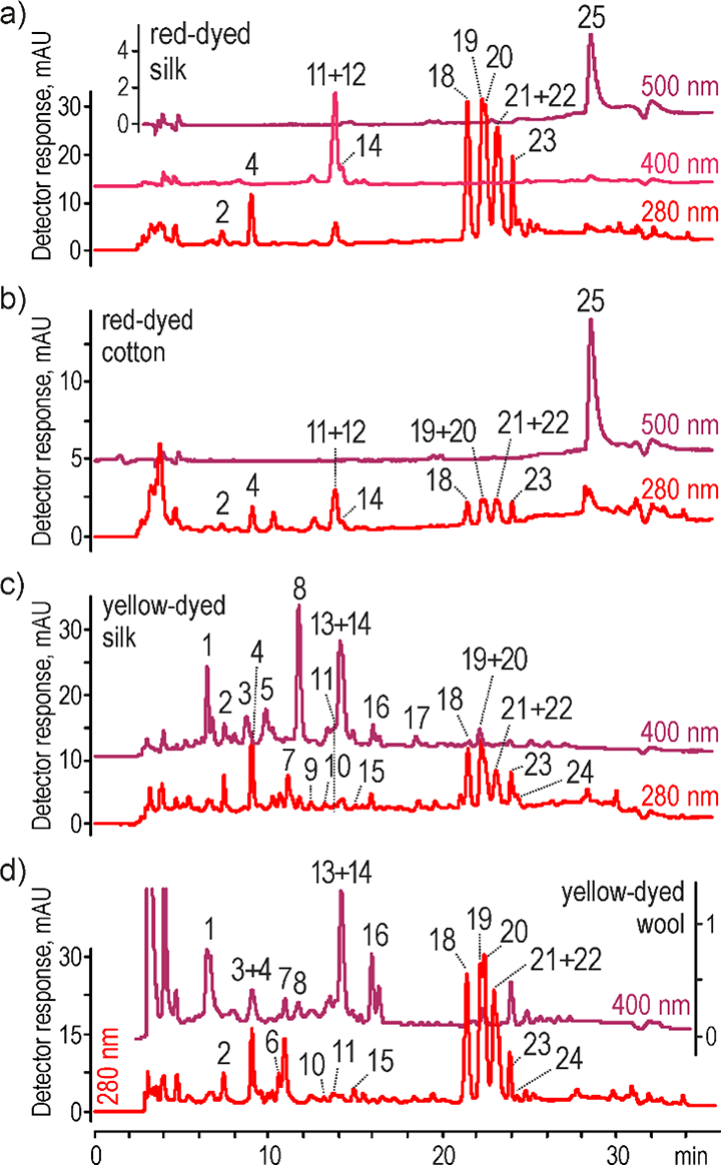
Chromatogram of extracts from safflower-dyed fibers acquired by UV–vis detector for (a) red-dyed silk, (b) red-dyed cotton, (c) yellow-dyed silk, and (d) yellow-dyed wool; peak numbers are decoded in Table 1.

The extracts of wool, silk, and cotton fibers experimentally dyed red and yellow with safflower (*Carthamus tinctorius* L.) were used to investigate safflower markers for their identification in historical textiles. The HPLC–UV–vis–ESI-MS analyses proved presence of several compounds. The chromatograms were acquired with a spectrophotometer at 280, 400, and/or 500 nm (Figure 1) and with a mass spectrometer using negative full scan and product ion modes. Commercially unavailable compounds were identified based on tandem mass spectrometry (MS/MS) experiments.

The deprotonated molecules ([M – H]^−^), chosen with spectrophotometric and full-scan mode MS chromatograms, were fragmented using triple quadrupole (and orbitrap) mass spectrometers with different collision energies. It led to identification of 10 quinochalcone *C-*glycosides and six noncolored safflower markers as well as nine other compounds. The identities of the ions and fragmentation pathways were determined using low- and high-resolution MS and MS/MS spectra.

Acquired data were included into the HPLC–UV–vis–ESI-MS/MS method intended for the identification of natural dyes in historical objects, antiques and works of art. It was used to study fibers taken from silk textiles dated from the 16th to 18th century and used in the vestments from the collections of Krakow churches.

### Quinochalcone *C*-Glycosides

Ten quinochalcone *C-*glycosides were identified in the safflower-dyed fibers. Their MS/MS spectra showed similarities regarding the presence of some ions and fragmentation pathways:

I. Ions common to MS/MS spectra of most quinochalcone *C-*glycosides:

- the *m*/*z* 119 (C_8_H_7_O) ion formed by cleavages of a *p*-coumaryl substituent next to a carbonyl group (a C7–C8 bond) *via* a remote hydrogen rearrangement;
- the *m*/*z* 145 (C_9_H_5_O_2_) ion formed by cleavages of a *p*-coumaryl substituent next to a carbonyl group (a C2–C7 bond) *via* a retro-heteroene reaction;
- the *m*/*z* 207 (C_7_H_11_O_7_) ion formed by the detachment of a C4 carbon (together with its substituents) and an adjacent hydroxyl group through a remote hydrogen rearrangement and an aromatic system reinstatement

II. Characteristic neutral losses formed *via* cleavages of a *C-*glycoside moiety:

- the 120 Da loss (C_4_H_8_O_4_) generated by a ^0,2^X cleavage of a *C-*glucose moiety (one of the characteristic fissions for quinochalcone homologues substituted by glucose at the C6 position^40^);
- the 150 Da loss (C_5_H_10_O_5_) generated by a ^0,1^X cleavage of a *C-*glucose moiety;
- the 163 Da loss (C_6_H_11_O_5_) - homolytic cleavage of a whole glucose moiety;
- the 90 Da loss (C_3_H_6_O_3_) generated by a ^0,1^X cleavage of a *C-*tetrose moiety or a ^0,2^X cleavage of a pentose moiety

III. Characteristic neutral losses formed *via* cleavages of a quinochalcone skeleton:

- the 120 Da loss (C_8_H_8_O), a molecule of 4-vinylphenol generated by a cleavage of a *p*-coumaryl substituent at a C7–C8 bond by a remote hydrogen rearrangement; according to the literature,^40^ a characteristic fission for quinochalcone homologues substituted by glucose at the C6 position;
- the 146 Da loss (C_9_H_6_O_2_) generated by breaking a *p*-coumaryl substituent at a C2–C7 bond by a γ-elimination or a retro-heteroene reaction;
- the 188 Da loss (C_11_H_8_O_3_) generated by the RDA reaction of quinone ring;
- the 208 Da loss (C_7_H_12_O_7_) formed through the elimination of a *C-*glucose moiety together with a C4 carbon and two hydroxyl groups (the one at a C4 carbon and another one substituted to an adjacent carbon atom) as a result of a remote hydrogen rearrangement and an aromatic system reinstatement.

**Figure 2:**
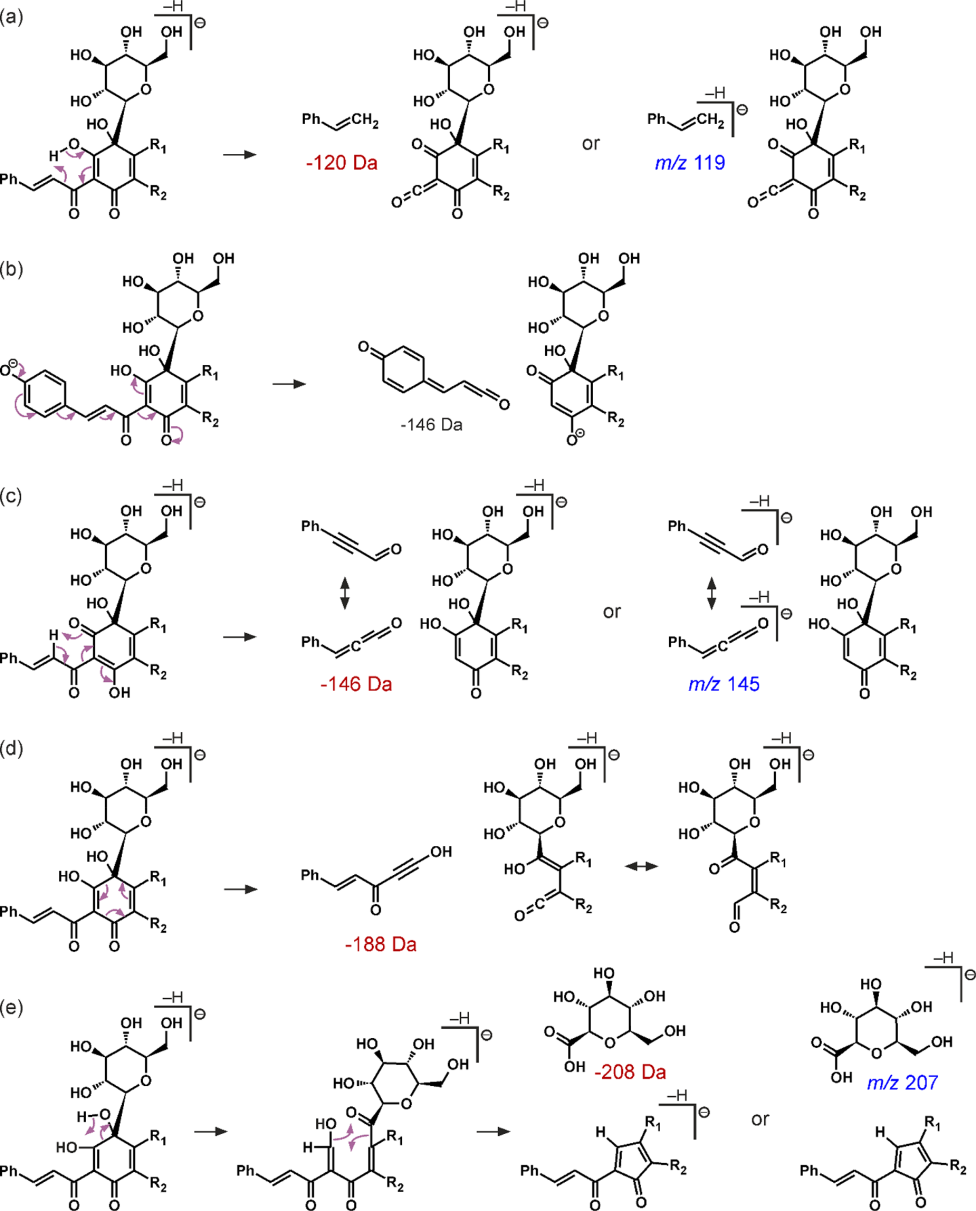
Proposed mechanisms of common fragmentations in quinochalcone *C*-glycosides through (a) a remote hydrogen rearrangement, (b) a γ-elimination, (c) a retro-heteroene reaction, (d) a RDA reaction, and (e) an aromatic system reinstatement preceded by a remote hydrogen rearrangement.

Thus, these ions and neutral losses can be used as markers for primary differentiation of quinochalcone *C-*glycosides. Proposed fragmentation mechanisms to support identification are presented in Figure 2.

**Figure 3:**
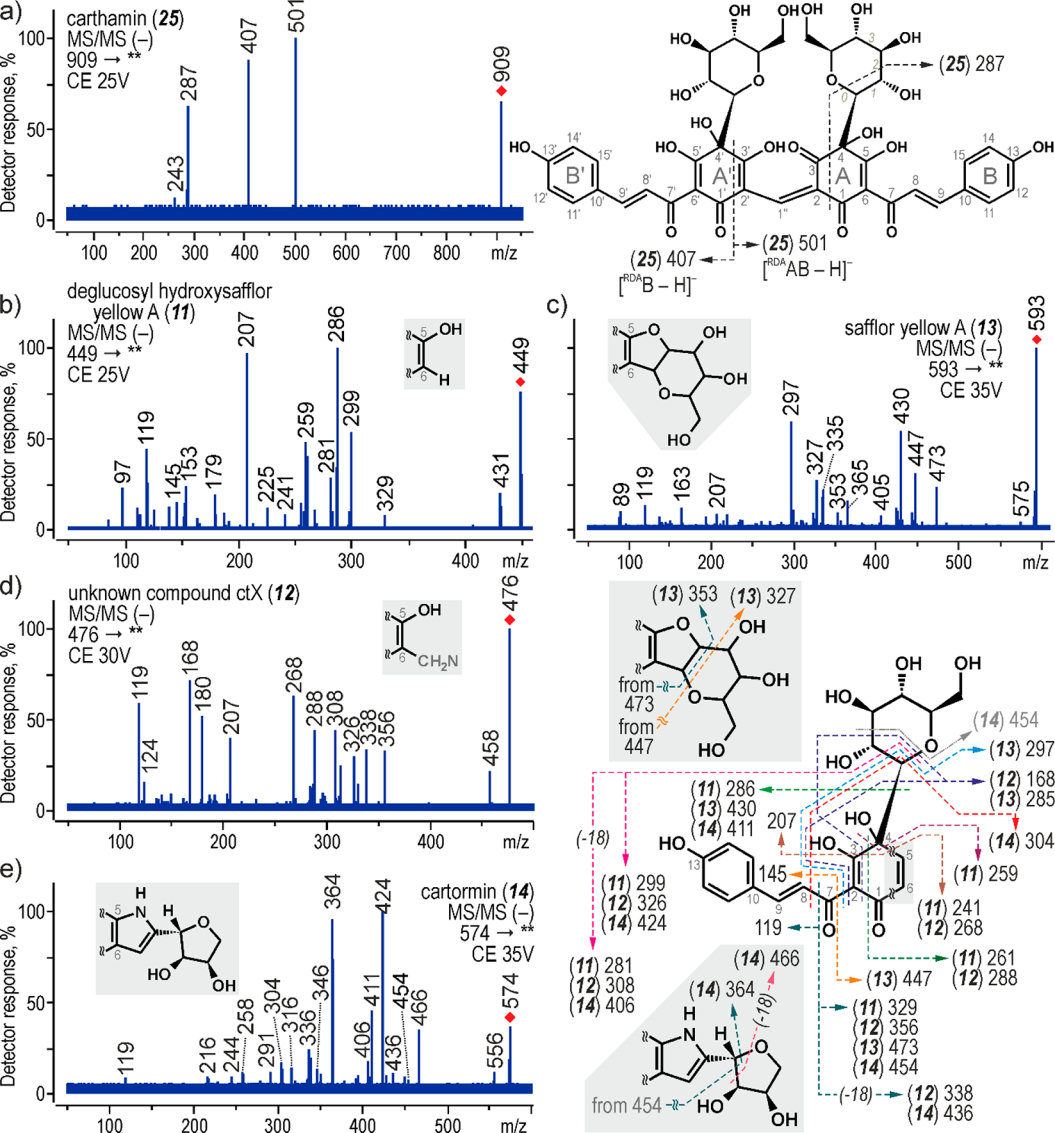
MS/MS spectra acquired by triple quadrupole MS in negative-ion mode and proposed fragmentation directions for (a) carthamin, (b) deglucosyl hydroxysafflor yellow A, (c) ctX, (d) safflor yellow A, and (e) cartormin

A peak corresponding to carthamin was not predominant in the chromatogram, and MS/MS spectra of its quasimolecular ion ([M – H]^−^) at *m*/*z* 909 were clear and simple with only few product ions. The first two at *m*/*z* 501 and 407 were formed *via* RDA reaction of quinone rings (A or A′) (Figure 3a). The resulting product ions can be coded as [^RDA^AB – H]^−^ and [^RDA^B – H]^−^, respectively. The A-ring fission accompanied by subsequent cross-ring cleavage of a glucose moiety (links numbered as 0 and 2, a ^0,2^X cleavage) resulted in formation of the *m*/*z* 287 ion, whereas further detachment of CO_2_ led to the signal of *m*/*z* 243. A proposed fragmentation pathway is fully reflected in the high-resolution MS/MS data of carthamin published previously without interpretation.^41^

Anhydrosafflor yellow B and its isomer gave deprotonated molecules [M – H]^−^ at *m*/*z* 1043, which generated only a few product ions (Supporting Information, Figure S2a,b). The *m*/*z* 1025 ion was formed by the loss of a H_2_O molecule from the parent ion, whereas the *m*/*z* 923 ion was a result of a detachment of C_4_H_8_O_4_ (120 Da) from a glucose moiety. The signals of *m*/*z* 449 and 593 were two complementary product ions generated by the cleavage of a C2′–C1″ bond, but the latter ion showed a very low intensity.

Due to the high molecular masses of carthamin and anhydrosafflor yellow B as well as the high stability of their deprotonated molecules (caused by the resonant structure of the double quinochalcone skeleton), their further decomposition needs collision energy higher than 45 V. As a consequence, the ions at *m*/*z* 207 and 119 were not present in the MS/MS spectra of carthamin and anhydrosafflor yellow B.

The other quinochalcone-*C*-glycosides showed common fragmentation pathways. Their MS/MS ions were formed *via* typical decomposition of a *C*-glucoside moiety and/or a quinochalcone skeleton. Moreover, losses of small molecules, such as H_2_O, CO, or CO_2_, were also observed. Detailed assignments of the product ions are presented in Table 2. The proposed identities were confirmed with the high-resolution data (Supporting Information, Table S1).

**Table 2:** MS/MS Ion Assignments of Quinochalcone *C*-Glycosides Present in Safflower-Dyed Fibers

no.	compd name	precursor ion, *m/z*	product ions, *m/z*	ion assignment
1	hydroxysafflor yellow A	611 [M – H]^−^	503	[M – H – C_3_H_6_O_3_ – H_2_O]^−^
			491	[M – H – C_4_H_8_O_4_]^−^
			473	[M – H – C_4_H_8_O_4_ – H_2_O]^−^
			403	[M – H – C_7_H_12_O_7_]^−^
			385	[M – H – C_7_H_12_O_7_ – H_2_O]^−^
			353	[M – H – 2C_4_H_8_O_4_ – H_2_O]^−^
			325	[M – H – 2C_4_H_8_O_4_ – CO – H_2_O]^−^
			313	[M – H – C_7_H_12_O_7_ – C_3_H_6_O_3_]^−^
			283	[M – H – C_7_H_12_O_7_ – C_4_H_8_O_4_]^−^
			207	[C_7_H_11_O_7_]^−^
			145	[C_9_H_5_O_2_]^−^
			119	[C_8_H_7_O]^−^
3	hydroxysafflor yellow B/C	611 [M – H]^−^	521	[M – H – C_3_H_6_O_3_]^−^
			445	[M – H – C_4_H_8_O_4_ – CO – H_2_O]^−^
			423	[M – H – C_11_H_8_O_3_]^−^
			403	[M – H – C_7_H_12_O_7_]^−^
			358	[M – H – C_3_H_6_O_3_ – C_6_H_11_O_5_]^•–^
			341	[M – H – C_4_H_8_O_4_ – C_5_H_10_O_5_]^−^
			313	[M – H – C_7_H_12_O_7_ – C_3_H_6_O_3_]^−^
			287	[^RDA^B – H – C_4_H_8_O_4_]^−^
			239	[M – H – C_7_H_12_O_7_ – C_8_H_8_O – CO_2_]^−^
			207	[C_7_H_11_O_7_]^−^
			119	[C_8_H_7_O]^−^
8 and 5	anhydrosafflor yellow B and anhydrosafflor yellow B isomer	1043 [M – H]^−^	1025	[M – H – H_2_O]^−^
			923	[M – H – C_4_H_8_O_4_]^−^
			593	[M – H – C_21_H_22_O_11_]^−^
			449	[M – H – C_27_H_30_O_15_]^−^
11	deglucosyl hydroxysafflor yellow A	449 [M – H]^−^	431	[M – H – H_2_O]^−^
			329	[M – H – C_8_H_8_O]^−^
			311	[M – H – C_4_H_8_O_4_ – H_2_O]^−^
			299	[M – H – C_5_H_10_O_5_]^−^
			286	[M – H – C_6_H_11_O_5_]^•–^
			259	[M – H – C_7_H_10_O_6_]^−^
			207	[C_7_H_11_O_7_]^−^
			179	[M – H – C_5_H_10_O_5_ – C_8_H_8_O]^−^
			153	[M – H – C_5_H_10_O_5_ – C_9_H_6_O_2_]^−^
			145	[C_9_H_5_O_2_]^−^
			119	[C_8_H_7_O]^−^
12	unknown compound (ctX)	476 [M – H]^−^	458	[M – H – H_2_O]^−^
			356	[M – H – C_8_H_8_O]^−^
			338	[M – H – C_8_H_8_O – H_2_O]^−^
			326	[M – H – C_5_H_10_O_5_]^−^
			308	[M – H – C_5_H_10_O_5_ – H_2_O]^−^
			288	[M – H – C_11_H_8_O_3_]^−^
			268	[M – H – C_7_H_12_O_7_]^−^
			207	[C_7_H_11_O_7_]^−^
			180	[M – H – C_5_H_10_O_5_ – C_9_H_6_O_2_]^−^
			168	[M – H – C_11_H_8_O_3_ – C_4_H_8_O_4_]^−^
			119	[C_8_H_7_O]^−^
13	safflor yellow A	593 [M – H]^−^	575	[M – H – H_2_O]^−^
			473	[M – H – C_8_H_8_O]^−^
			447	[M – H – C_9_H_6_O_2_]^−^
			430	[M – H – C_6_H_11_O_5_]^•–^
			405	[M – H – C_11_H_8_O_3_]^−^
			365	[M – H – C_8_H_8_O – C_3_H_6_O_3_ – H_2_O]^−^
			353	[M – H – C_8_H_8_O – C_4_H_8_O_4_]^−^
			335	[M – H – C_8_H_8_O – C_4_H_8_O_4_ – H_2_O]^−^
			327	[M – H – C_9_H_6_O_2_ – C_4_H_8_O_4_]^−^
			297	[M – H – C_9_H_6_O_2_ – C_5_H_10_O_5_]^−^
			207	[C_7_H_11_O_7_]^−^
			119	[C_8_H_7_O]^−^
14	cartormin	574 [M – H]^−^	556	[M – H – H_2_O]^−^
			466	[M – H – C_3_H_6_O_3_ – H_2_O]^−^
			454	[M – H – C_8_H_8_O]^−^
			436	[M – H – C_8_H_8_O – H_2_O]^−^
			424	[M – H – C_5_H_10_O_5_]^−^
			411	[M – H – C_6_H_11_O_5_]^•–^
			406	[M – H – C_5_H_10_O_5_ – H_2_O]^−^
			364	[M – H – C_4_H_8_O_4_ – C_3_H_6_O_3_]^−^
			346	[M – H – C_8_H_8_O – C_3_H_6_O_3_ – H_2_O]^−^
			338	[M – H – C_4_H_8_O_4_ – C_3_H_6_O_3_ – C_2_H_2_]^−^
			336	[M – H – C_4_H_8_O_4_ – C_3_H_6_O_3_ – CO]^−^
			316	[M – H – C_4_H_8_O_4_ – C_8_H_8_O – H_2_O]^−^
			304	[M – H – C_8_H_8_O – C_5_H_10_O_5_]^−^
			291	[M – H – C_8_H_8_O – C_6_H_11_O_5_]^•–^
			244	[M – H – C_4_H_8_O_4_ – C_3_H_6_O_3_ – C_8_H_8_O]^−^
			216	[M – H – C_4_H_8_O_4_ – C_8_H_8_O – C_3_H_6_O_3_ – CO]^−^
			119	[C_8_H_7_O]^−^
16	safflomin C	613 [M – H]^−^	595	[M – H – H_2_O]^−^
			551	[M – H – H_2_O – CO_2_]^−^
			533	[M – H – 2H_2_O – CO_2_]^−^
			431	[M – H – C_8_H_8_O – H_2_O – CO_2_]^−^
			425	[M – H – C_11_H_8_O_3_]^−^
			407	[^RDA^B – H]^−^
			361	[M – H – C_7_H_12_O_7_ – CO_2_]^−^
			317	[M – H – C_7_H_12_O_7_ – 2CO_2_]^−^
			287	[^RDA^B – H – C_4_H_8_O_4_]^−^
			241	[M – H – C_7_H_12_O_7_ – C_8_H_8_O – CO_2_]^−^
			207	[C_7_H_11_O_7_]^−^
			145	[C_9_H_5_O_2_]^−^
			119	[C_8_H_7_O]^−^
25	carthamin	909 [M – H]^−^	501	[^RDA^AB – H]^−^
			407	[^RDA^B – H]^−^
			287	[^1,3^AB – H – C_4_H_8_O_4_]^−^
			243	[^1,3^AB – H – C_4_H_8_O_4_ – CO_2_]^−^

Two colorants, eluted at *t*_R_ 6.5 and at 9.0 min, had the same value of their [M – H]^−^ ions at *m*/*z* 611, but their MS/MS spectra differed from each other (Supporting Information, Figure S2c,d). They were identified to be isomers, hydroxysafflor yellow A and hydroxysafflor yellow B or C, respectively. The first compound showed one of the product ions at *m*/*z* 491 correlating with the loss of a C_4_H_8_O_4_ molecule (120 Da), while the second compound containing a pentose at the C6 position lost a C_3_H_6_O_3_ molecule (90 Da) as a result of a ^0,2^X cleavage. Moreover, the signal at *m*/*z* 287 was formed in analogous way, as was the case of carthamin *via* RDA reaction of quinone ring accompanied by cross-ring cleavage of a glucose moiety.

The MS/MS spectra (Figure 3b) of the *m*/*z* 449 ion ([M – H]^−^, *t*_R_ 13.3 min) showed an analogous fragmentation pathway. Thus, this compound was determined to be deglucosyl hydroxysafflor yellow A, presumably a degradation product of carthamin. Safflower yellow A (*t*_R_ 13.7 min) gave the [M – H]^−^ ion at *m*/*z* 593. Its MS/MS spectra (Figure 3c) showed several product ions that were congruous with the spectrum presented in the literature.^42^ The next compound, safflomin C (*t*_R_ 16.0 min), was identified on the basis of the [M – H]^−^ ion at *m*/*z* 613 and its characteristic fragmentation (the MS/MS spectrum is shown in the Supporting Information, Figure S2e), which was consistent with previously published data.^40^ The ion at *m*/*z* 407 was formed in consequence of the RDA reaction of a quinone ring and decomposition of C1–C6 and C4–C5 bonds (the ion marked as [^RDA^B – H]^−^), whereas 287 corresponded to the ion following the further loss of a C_4_H_8_O_4_ molecule. The last two colorants showed odd values of their [M – H]^−^ ions (*m*/*z* 476 for the compound eluted at 13.5 min and *m*/*z* 574 for the one at 13.9 min), thus indicating the presence of a nitrogen atom in the molecular structures. The MS/MS spectra showed that their fragmentation was partially similar to the fragmentation of other quinochalcone *C-*glycosides, and the only differences in their structures concerned parts attached to a quinochalcone skeleton in the C5–C6 position (where also nitrogen atom was located). Although the formula of the first compound (ctX, [M – H]^−^ at *m*/*z* 476) was determined to be C_22_H_23_O_11_N (Supporting Information, Table S1), its exact structure remained unconfirmed as the nitrogen-containing part did not show any particular fragmentation (Figure 3d). However, both ions at *m*/*z* 207 and 268 (formed through a remote hydrogen rearrangement and aromatic system reinstatement) were very intense, which, according to previous studies,^42^ indicated the presence of a hydroxyl group in the C5 position and, as a consequence, also a CH_2_N moiety in a C6 substituent. An ion formula of the second colorant ([M – H]^−^ at *m*/*z* 574) was determined from an accurate *m*/*z* value to be C_27_H_28_O_13_N, which matched that of cartormin. The identification was also confirmed by its 2-fold fragmentation. On one hand, the MS/MS spectrum (Figure 3e) showed signals formed by a typical decomposition of quinochalcone *C-*glycosides, but on the other hand, an alternative fragmentation pathway was also observed. The signals at *m*/*z* 466, 364, 346, 338, and 244 were related to a ^0,1^X cleavage of a tetrose moiety in a C5–C6 substituent, resulting in the loss of a C_3_H_6_O_3_ molecule.

### Other Markers

The subsequent set of peaks (Figure 1) was characterized by deprotonated molecules with the same *m*/*z* of 582. These peaks corresponded to a series of six compounds, probably isomers, eluting one by one at *t*_R_ 21.4, 22.3, 22.5, 23.1, 23.3, and 24.1 min and coded as ct1, ct2, ct3, ct4, ct5, and ct6. Some of them (ct2 and ct3 as well as ct4 and ct5) were coeluted with each other, so they were observed in the chromatogram as four chromatographically distinct peaks. Although these ct-components, unlike quinochalcone *C-*glycoside, do not absorb radiation in visible range,^8^ they resist strong mineral acids and photodegradation processes; thus, they have been proposed as excellent markers to identify safflower red, even in discolored historical samples or in highly hydrolyzed extracts.^10^ Despite the general fact about an odd number of nitrogen atoms in the formula (due to an even *m*/*z*-value (582) of the deprotonated molecule), the chemical structures of the six ct components, to the best of our knowledge, had not hitherto been reported. Nevertheless, these compounds have been allotted as the basic safflower markers.^10^

**Figure 4:**
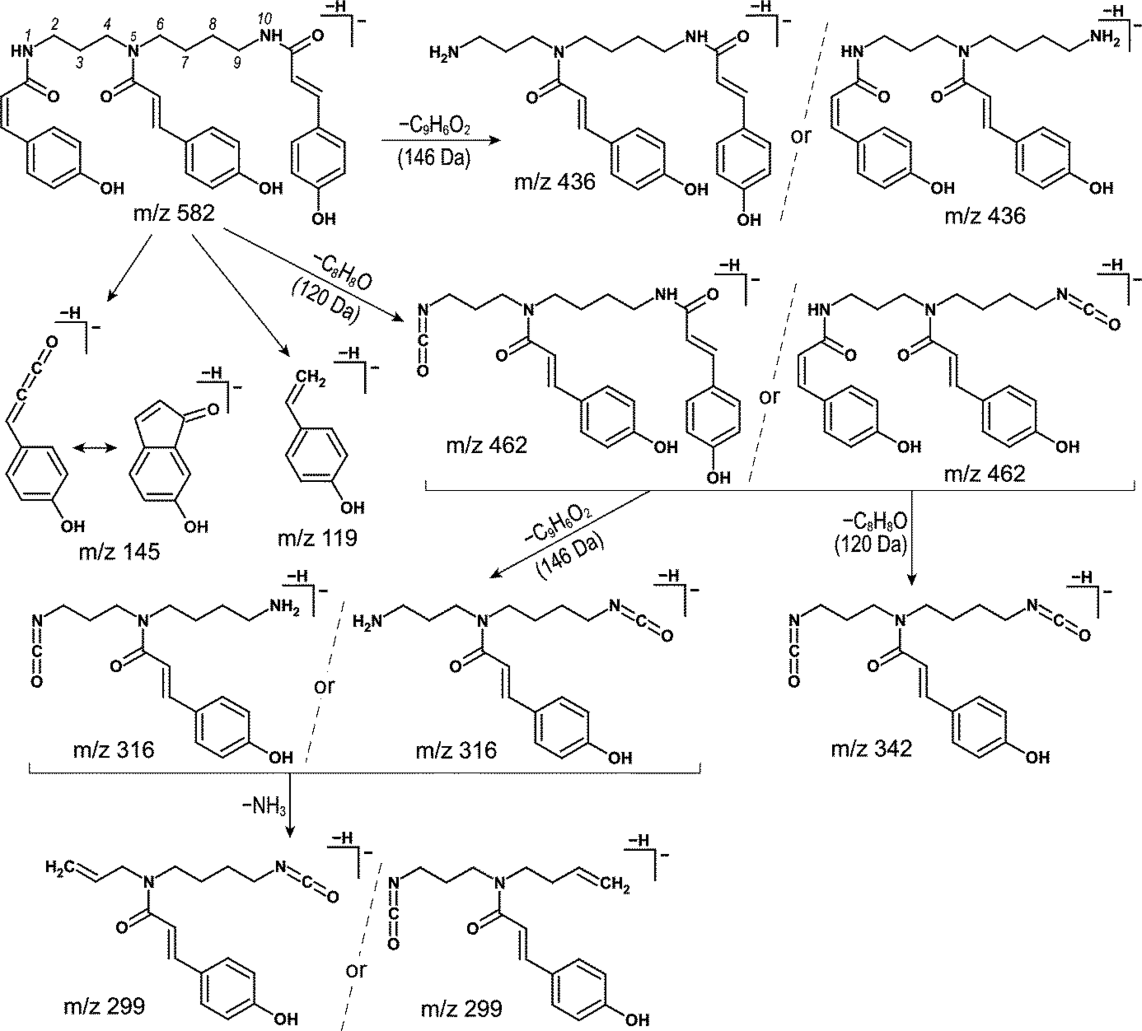
Proposed fragmentation pathway of ct1–ct6 compounds on the example of *N*^1^*,N*^5^*,N*^10^-tri-*p*-(*ZEE*)-coumaroylspermidine in negative-ion mode.

MS/MS spectra acquired for deprotonated molecules at *m*/*z* 582 of the ct compounds were almost identical (Supporting Information, Figure S3), only slightly different in intensity of the product ions. They were rather simple, with only a few intensive and clear signals. The two main losses were observed: a detachment of 120 Da *p*-hydroxystyrene (C_8_H_8_O) and elimination of 146 Da 6-hydroxy-1-indenone (C_9_H_6_O_2_). Thus, the major two ions at *m*/*z* 462 and 342 were formed by the loss of one or two 120 Da molecules ([M – H – C_8_H_8_O]^−^ and [M – H – 2C_8_H_8_O]^−^), whereas signals at *m*/*z* 436 and 316 corresponded to [M – H – C_9_H_6_O_2_]^−^ and [M – H – C_9_H_6_O_2_ – C_8_H_8_O]^−^, respectively. Furthermore, a *p*-coumaryl group was a source of signals at *m*/*z* 119 and 145 (typical ones for this substituent, as has been given above for quinochalcone *C-*glycosides). All this information together led to conclusion that each ct compound represented one of eight different stereoisomeric forms of *N*^1^*,N*^5^*,N*^10^-tri-*p*-coumaroylspermidine (*ZZZ*, *ZZE*, *ZEZ*, *EZZ*, *ZEE*, *EZE*, *EEZ*, and *EEE*) which vary in the geometry of three existing olefinic double bonds (proposed fragmentation pathway is showed in Figure 4). This identification was consistent with the data reported previously.^43,44^ Moreover, it was also confirmed by the high-resolution data acquired with Orbitrap MS (Supporting Information, Table S1). The deprotonated molecule formulas of the ct compounds were calculated to be C_34_H_36_O_6_N_3_, whereas the most intense MS/MS signals at *m*/*z* 462, 436, 342, 316, and 299 were determined to be C_26_H_28_O_5_N_3_, C_25_H_30_O4N_3_, C_18_H_20_O_4_N_3_, C_17_H_22_O_3_N_3_, and C_17_H_19_O_3_N_2_, respectively.

Considering the UV spectra published for ct components^8,10^ and *N*^1^*,N*^5^*,N*^10^-tri-*p*-coumaroylspermidines,^43^ the ZZZ isomer eluted first (ct1) from a reversed-phase column as the first peak, whereas the *EEE* isomer was the last one (ct6). Compounds eluted between them were a mixture of different *E*/*Z* isomers. Their elution order is a result of differences in their overall polarity because the dipole moments of these isomers change while the stereoisomeric form of *p*-coumaroylspermidine moieties changes.

Since the number of present *N*^1^*,N*^5^*,N*^10^-tri-*p*-coumaroylspermidine isomers results from their ability to photoisomerize,^43,45^ the intensity of each individual ct peak may differ depending on the different irradiance flux densities received by safflower-dyed samples.

Apart from quinochalcones and *N*^1^*,N*^5^*,N*^10^-tri-*p*-coumaroylspermidine isomers, two flavonols and four flavonol glycosides were found in safflower extracts. They were identified to be quercetin, quercetin *O-*hexoside ([M – H]^−^ at *m*/*z* 463 fragmenting to 301, 300, 271), hyperoside, rutin, kaempferol *O-*deoxyhexosylhexoside ([M – H]^−^ at *m*/*z* 593 fragmenting to 285), and apigenin. Moreover, *p*-hydroxybenzoic acid ([M – H]^−^ at *m*/*z* 137 fragmenting to 93), *p*-hydroxystyrene ([M – H]^−^ at *m*/*z* 121 fragmenting to 77), and *p*-coumaric acid were found, maybe as a degradation product of quinochalcones and *N*^1^*,N*^5^*,N*^10^-tri-*p*-coumaroylspermidines.

### Presence of Colorants in Yellow- and Red-Dyed Fibers

The chromatograms registered by a spectrophotometric detection (Figure 1) showed more complex composition of the extracts obtained for yellow-dyed fibers (silk and wool) than for red-dyed fibers (silk and cotton). Nevertheless, all extracts contained cartormin, deglucosyl hydroxysafflor yellow A, and *N*^1^*,N*^5^*,N*^10^-tri-*p*-coumaroylspermidine isomers as well as *p*-hydroxybenzoic acid and *p*-hydroxystyrene (Table 1). Deglucosyl hydroxysafflor yellow A probably was produced as a result of the degradation or hydrolysis of carthamin, precarthamin, anhydrosafflor yellow B, and/or safflor yellow B, whereas *p*-hydroxybezoic acid and *p*-hydroxystyrene were formed from a chalcone skeleton or from *p*-coumaric acid moieties. Carthamin, similar to ctX, was prevalent only in the extracts of red-dyed fibers. It may suggest that ctX was synthesized during the dyeing process due to the high pH or by reaction of carthamin or other precursors with ammonia. Thus, this compound may be one of the markers for safflower red, directly related to the dyeing method. Other quinochalcone *C-*glycosides, flavonoid glycosides, flavonoids, and *p*-coumaric acid were found only in the yellow-dyed fibers.

### Analysis of Historical Samples

The most intense precursor and product ion pairs (transitions) of the identified safflower markers were used to expand the HPLC–UV–vis–ESI-MS/MS method developed prior in dynamic multiple reaction monitoring (dMRM) mode.^29,30^ All colorants that were found in historical samples and others not presented here have been characterized in previous publications.^29,30^ Since analytes were only monitored while they were being eluted from the LC (during the retention window), the dMRM mode provided superior sensitivity and selectivity for targeted compounds in complex samples maximizing the detection capability of the MS.

The developed HPLC–UV–vis–ESI-MS/MS method was applied to investigate natural dyes in silk fibers accordingly to the analytical protocol proposed previously.^30^ The thread samples were taken from 16th to 18th century textiles used in the vestments owned by Krakow churches. The samples, identified compounds, and dyes are presented in Table 3.

**Table 3:** Compounds and Dyes Identified in Silk Textiles Dated from 16th to 18th Century^a^

church	textile no.^c^	object^c^	origin and dating^c^	fiber color	identified compounds	original dye	remarks
Church of St. Andrew (Monastery Church of the Poor Clares)	no. 179	dalmatic, textile on back and on front sides	Europe, the 17th cent.^d^	pinkish orange	fustin^b^, *p*-coumaric acid, **carminic acid**^b^, **deglucosyl hydroxysafflor yellow A**, luteolin 7-*O-*glucoside^b^, hesperidin^b^, ctZ, eriodictyol^b^, **diosmin**^b^, **sulfuretin**^b^, luteolin^b^**ct1**, **ct2**, **ct3**, **ct4**, **ct5**, **ct6**, **diosmetin**^b^carthamin	safflower + traces of young fustic, weld, and cochineal (of unknown origin)	
Church of St Francis of Assisi (Monastery Church of the Franciscans)	no. 262	chasuble, textile from sides	Europe, the 17th cent.^e^	pinkish orange	**ctY**, **deglucosyl hydroxysafflor yellow A**, ctX, hesperidin^b^**ctZ**, diosmin^b^**ct1**, **ct2**, **ct3**, **ct4**, **ct5**, **ct6**, **carthamin**	safflower	
Church of St. Peter and St. Paul	no. 277	chasuble, main textile	Europe, the 17th cent.^e^	bright red	brazilein^b^protosappanin B^b^caes1^b^**caesD**^b^carminic acid^b^deglucosyl hydroxysafflor yellow A, **urolithin C**^b^hesperidin^b^caes2^b^**ct1**, **ct2**, **ct3**, **ct4**, **ct5**, **ct6**, β/γ-aminoorcein^b^β/γ-aminoorceinimine^b^β/γ-aminoorcein^b^**α-aminoorcein**^b^β/γ-hydroxyorcein^b^**α-hydroxyorcein**^b^	safflower + sappanwood + orchil	presence of synthetic dye, original fiber color - red or orange
Basilica of Holey Trinity (Monastery Church of the Dominicans)	no. 285	chalice velum, main textile	Europe, the 17th cent.^e^	bright red	brazilein^b^**protosappanin B**^b^caes1^b^**caesD**^b^*p*-coumaric acid, carminic acid^b^deglucosyl hydroxysafflor yellow A, **urolithin C**^b^**ct1**, **ct2**, **ct3**, **ct4**, **ct5**, **ct6**, carthamin	safflower + sappanwood	presence of synthetic dye, original fiber color - orange
Church of the Visitation of the Blessed Virgin Mary (Monastery Church of the Carmelites)	no. 325	chasuble, textile from sides	Near East, the 16th cent.^f^	blueish green	**hydroxysafflor yellow A**, *p*-coumaric acid, **deglucosyl hydroxysafflor yellow A**, safflomin C, **ctZ**, **ct1**, **ct2**, **ct3**, **ct4**, **ct5**, **ct6**, isatin^b^**indigotin**^b^indirubin^b^carthamin	safflower + indigo	
Church of St. Barbara	no. 329	chasuble, main textile	Near East, the 17th cent.^f^	pinkish orange	*p*-coumaric acid, **deglucosyl hydroxysafflor yellow A**, **ct1**, **ct2**, **ct3**, **ct4**, **ct5**, **ct6**, carthamin	safflower	
Church of St. Michael the Archangel and St. Stanislaus Bishop and Martyr (Monastery Church of the Pauline Fathers)	no. 334	chasuble, orphrey and around-neck-opening textile	Near East, the 17th cent.^f^	dark green	**hydroxysafflor yellow A**^b^*p*-coumaric acid, **deglucosyl hydroxysafflor yellow A**, **kaempferol*****O-*****deoxyhexosyl-hexoside**, kaempferol 3-*O-*glucoside^b^**ctZ**, **ct1**, ct2, ct3, ct4, ct5, ct6, isatin^b^**indigotin**^b^indirubin^b^	safflower + indigo	
Church of the Annunciation to the Blessed Virgin Mary (Monastery Church of the Capuchins)	no. 336	chalice velum, main textile	Near East, the 17th cent.^f^	orange	**brazilein**^b^**protosappanin B**^b^caes1^b^**caesD**^b^*p*-coumaric acid, carminic acid^b^deglucosyl hydroxysafflor yellow A, **urolithin C**^b^kaempferol *O-*deoxyhexosyl-hexoside^b^kaempferol 3-*O-*glucoside^b^**caes2**^b^ctZ, **ct1**, **ct2**, **ct3**, **ct4**, **ct5**, **ct6**	safflower + sappanwood	
Church of the Annunciation to the Blessed Virgin Mary (Monastery Church of the Capuchins)	no. 338	chasuble, around-neck-opening textile	Near East, the 17th cent.^f^	orange	**brazilein**^b^**protosappanin B**^b^caes1^b^**caesD**^b^hyperoside^b^deglucosyl hydroxysafflor yellow A, **urolithin C**^b^kaempferol 3-*O-*glucoside^b^**caes2**, **ct1**, **ct2**, **ct3**, **ct4**, **ct5**, **ct6**	safflower + sappanwood	
Church of St. Barbara	N/A (KsB-9^g^)	chalice velum, main textile	Europe, the 18th cent.^f^	pinkish orange	*p*-coumaric acid, **deglucosyl hydroxysafflor yellow A**, **ctZ**, **ct1**, **ct2**, **ct3**, **ct4**, **ct5**, **ct6**, curcumin^b^	safflower + trace of turmeric	
Church of St. Joseph (Monastery Church of the Bernardine Nuns)	N/A (B-4^g^)	chasuble, back orphrey	Near East, the 18th cent.^f^	pinkish orange	carminic acid^b^luteolin 7-*O-*glucoside^b^hesperidin^b^ctY, **deglucosyl hydroxysafflor yellow A**, cartormin, kaempferol *O-*deoxyhexosyl-hexoside, ellagic acid^b^apigenin 7-*O-*glucoside^b^**ctZ**, diosmin^b^luteolin^b^**ct1**, **ct2**, **ct3**, **ct4**, **ct5**, **ct6**, isatin^b^indigotin^b^carthamin, **bixin**^b^	safflower + annatto + traces of indigo/woad	

a **Bold and underlined**, the main compounds; **bold**, the secondary compounds; nonhighlighted, the minor compounds; ct1–ct6, *E/Z* stereoisomers of *N*^1^*,N*^5^*,N*^10^-tri-*p*-coumaroylspermidine.

b Identified previously and depicted in refs (29 and 30).

c Detailed data presented in “*KATALOG TKANIN z zasobów kościelnych Krakowa z czasów od XV do końca XVII*” (in Polish); Krupa, N., Ed.; Kraków (in printing).

d Textile origin and dating have been established by A. Warzecha.

e Textile origin and dating have been established by K. Moskal.

f Textile origin and dating have been established by B. Biedrońska-Słota.

g Initial object number.

Safflower (*Carthamus tinctorius* L.) was a dye identified in 11 thread samples taken from silk textiles which, according to art historians, mostly date back to the 17th century. Six of them originate from the Near East and the other five from Europe. All of the extracts contained all ct compounds and deglucosyl hydroxysafflor yellow A, sometimes also hydroxysafflor yellow A or kaempferol *O-*deoxyhexosylhexoside. Although carthamin is a water-insoluble red compound, its significant amount was found in the extract of one yellow thread (no. 262) and only trace amounts in five other samples. This proves that, even though very sensitive, carthamin exposed to formic acid does not hydrolyze under the extraction conditions.

Apart from already identified safflower markers, two new quinochalcone *C-*glycosides were found in historical samples (coded ctY and ctZ). Both of them were clearly visible in the 400 nm chromatograms. CtY (*t*_R_ 12.9 min) was present in two (nos. 262 and B-4) and ctZ (*t*_R_ 17.4 min) in seven out of 11 samples (nos. 179, 262, 325, 334, 336, KsB-9, and B-4). Their presence may be related to the dyeing method or degradation process, as reported previously.^35^ Thus, the presence of a ctY compound may be a result of the specific dyeing-red conditions or photodegradation of carthamin, which is accompanied by the ctY compound formation (the respective studies are underway and will be published soon).

**Figure 5:**
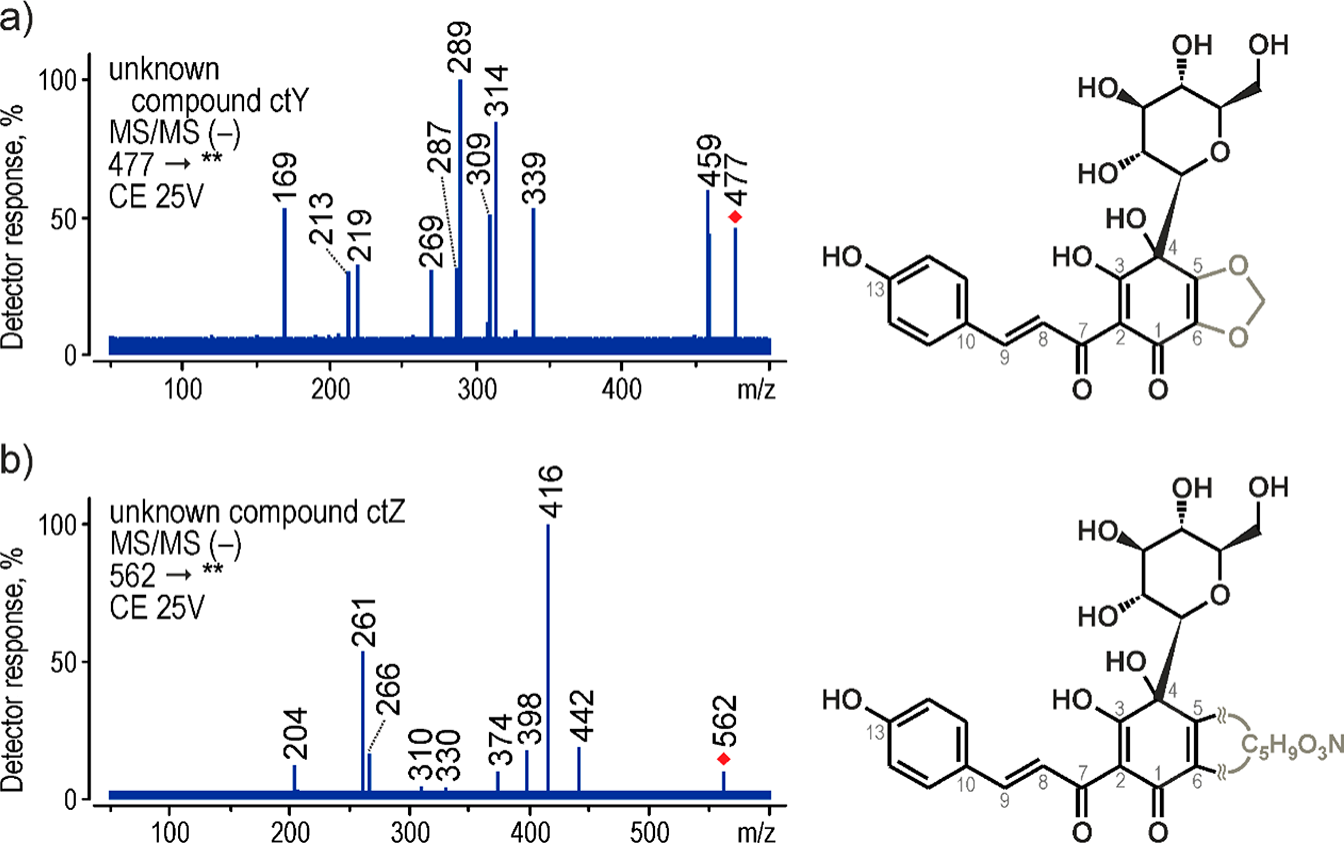
MS/MS spectra acquired by triple quadrupole MS in negative-ion mode and proposed structures for (a) ctY and (b) ctZ.

CtY generated the [M – H]^−^ ion at *m*/*z* 477. Its fragmentation pathway was very similar to the *m*/*z* 476 ion (ctX). The MS/MS spectrum (Figure 5a) showed ions at *m*/*z* 459 [M – H – H_2_O]^−^, 339 [M – H – H_2_O – C_4_H_8_O_4_]^−^, 327 [M – H – C_5_H_10_O_5_]^−^, 314 [M – H – C_6_H_11_O_5_]^•–^, 309 [M – H – H_2_O – C_5_H_10_O_5_]^−^, 289 [M – H – C_11_H_8_O_3_]^−^, 269 [M – H – C_7_H_12_O_7_]^−^, and 169 [M – H – C_11_H_8_O_3_ – C_4_H_8_O_4_]^−^. All these signals were exactly 1 Da higher than the ones in the ctX spectrum. However, since the *m*/*z* 207 ion was very low and rather acquired at higher collision energy, it could be assumed that ctY did not have a hydroxyl group in the C5 position. Thus, considering its ion formula, that according to the accurate mass measurement was determined to be C_22_H_21_O_12_, it was assumed that the C5–C6 carbons most likely formed part of a cyclic structure of 1,3-dioxolone.

The second colorant, ctZ, gave the deprotonated molecule at *m*/*z* 562. Its MS/MS spectra showed typical fragmentation for quinochalcone *C-*glycosides (Figure 5b). The *m*/*z* 442 ion was generated by detachment of 4-vinylphenol (C_8_H_8_O), and the *m*/*z* 374 ion was produced through the RDA reaction. The most intense signal at *m*/*z* 416, however, was formed by the loss of 146 Da (C_9_H_6_O_2_), and all sequential major ions, including 398, 266, and 261, resulted from its further fragmentation. The former two corresponded with [M – H – C_9_H_6_O_2_ – H_2_O]^−^ and [M – H – C_9_H_6_O_2_ – C_5_H_10_O_5_]^−^, respectively, while the last one was generated through the RDA reaction by cleaving the C4–C5 and C1–C6 bonds. Even though high-resolution Orbitrap MS determined the *m*/*z*-562-ion formula to be C_26_H_28_O_13_N, the structure of ctZ remained unconfirmed. However, since the *m*/*z* 207 ion was very low, it was assumed that there was no hydroxyl group in a ctZ molecule, and as most quinochalcone *C-*glycosides, it differed only in the C5–C6 part. The general formula of this part was C_5_H_9_O_3_N.

**Figure 6:**
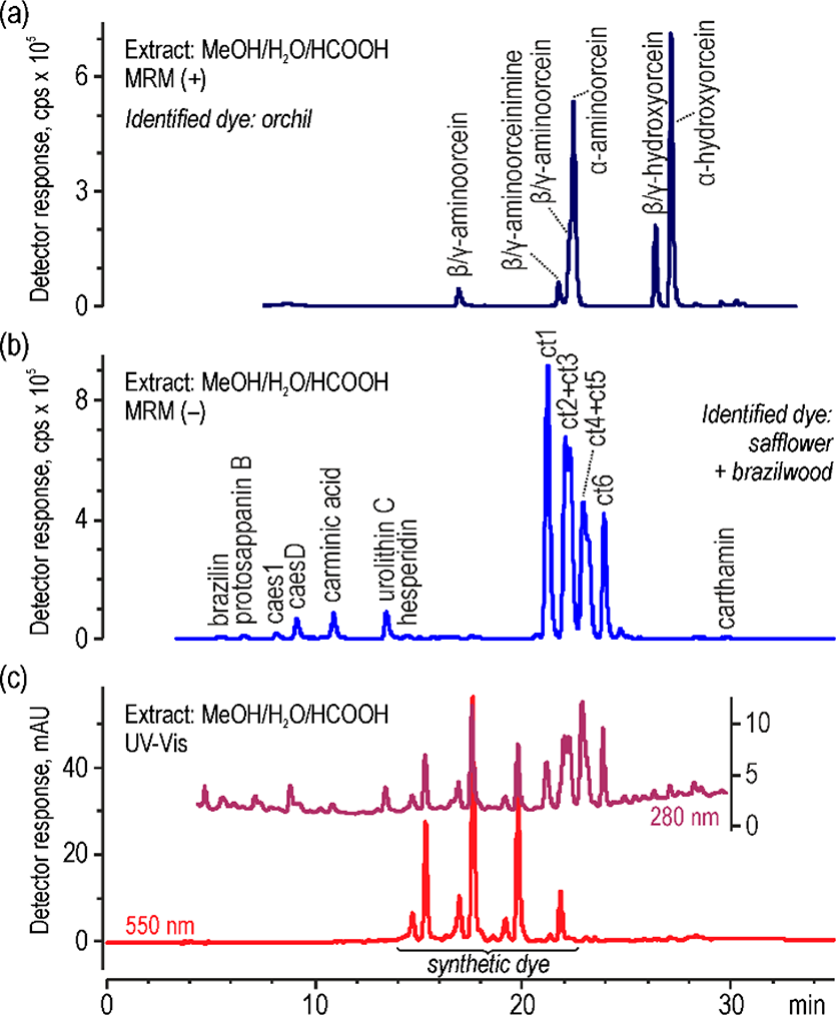
Chromatograms of red thread extract (textile no. 277) acquired by MS detector in positive dMRM mode (a), in negative dMRM mode (b), and by UV–vis detector (c).

In Asia and Europe, *Carthamus tinctorius* L. was not only a source of red, but also yellow or orange,^1,2^ as was found for most of the analyzed fibers. This was achieved by using a simple water bath in which only yellow chalcones were released from petals. Thus, safflower was used individually to dye two samples, one yellow (no. 262) and one orange with a red hue (no. 329); however, much often it was applied in a configuration with other dyes. The results showed that three out of 11 examined threads (nos. 285, 336, and 338) were dyed with a combination of safflower and sappanwood. This mixture resulted in an orange color to the yarns, but one of them (no. 285) was redyed with a red synthetic dye. The same synthetic dyes were also found in another thread sample (no. 277) that originally was dyed with a ternary mixture of safflower, sappanwood, and orchil (Figure 6). Since this combination of dyes was only used in this one sample, the color of the thread was difficult to definitely define; possibly it was dark orange or even pink with an orange hue. Moreover, another three orange samples (nos. 179, KsB-9, and B-4) were dyed by a mixture of safflower with young fustic/weld/unknown cochineal, turmeric, and annatto, respectively.

**Figure 7:**
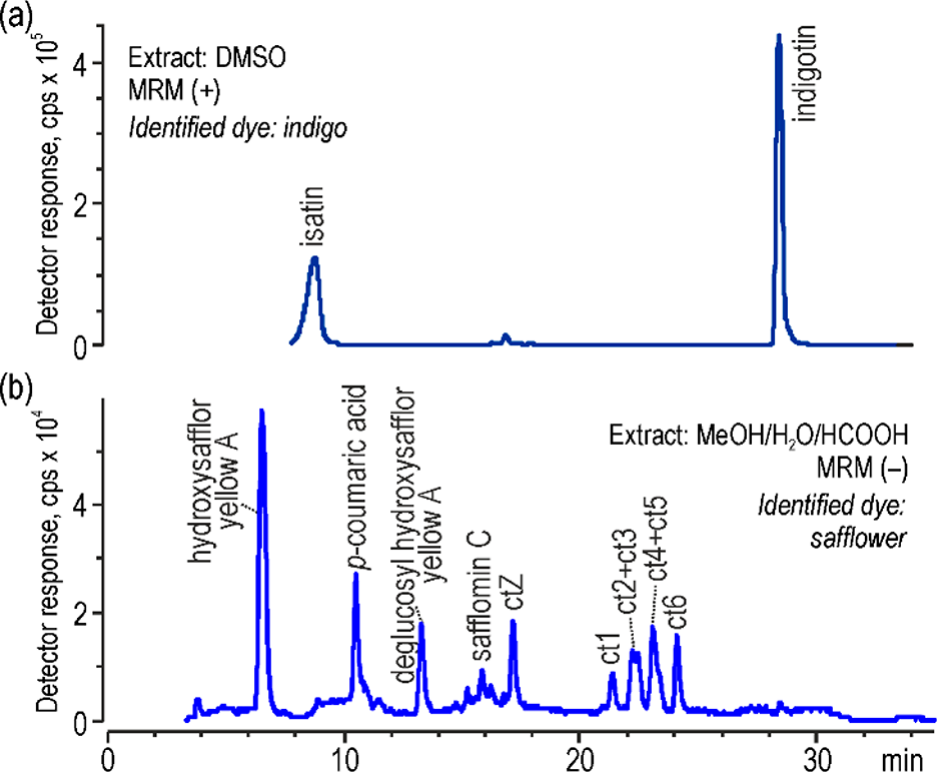
Chromatograms of green thread extracts (textile no. 325) acquired by MS detector in negative dMRM mode (a) and by UV–vis detector (b).

Safflower yellow also was used together with indigo to dye green.^2^ In fact, both dyes were identified in two 16th and 17th century thread samples (no. 325 and no. 334, respectively) originated from the Near East. Although the provenance of indigo dye could not be established by this research, it can be assumed, given the origin of the textiles, that probably *Indigofera* plants were a source of indigo. Interestingly, in both these samples the signals of hydroxysafflor yellow A and deglucosyl hydroxysafflor yellow A were more intense than the signals of ct compounds (Figure 7), contrary to the previous samples. Since they are possible decomposition products of safflor yellow B and carthamin, their presence may result from different conditions of dyeing green. Moreover, the extract of the no. 325 thread, as the only historical sample, involved also safflomin C.

## Conclusions

The result presented in this paper provide a new knowledge regarding the tandem mass spectrometric detection of safflower components. A detailed and comprehensive investigation on yellow and red safflower-dyed fibers (silk, cotton, and wool) was conducted with the use of HPLC–UV–vis–ESI-MS/MS and UPLC–HESI-HRMS/MS. The findings enabled to characterize the composition of the color compounds, including safflower markers, present in the orange, pink, red, and green threads sampled from 16th to 18th century silk textiles of European and Near Eastern origin.

The MS/MS studies leaded to the structure elucidation of six colorless markers, which proved to be *E*/*Z* stereoisomers of *N*^1^,*N*^5^,*N*^10^-tri-*p*-coumaroylspermidine (formerly known as ct compounds), and to the characterization of other compounds (including ten quinochalcones) extracted from dyed fibers. The chemical compositions, identities, and proposed fragmentation pathways were confirmed by high-resolution mass spectrometry data acquired by Orbitrap MS. As a consequence, the high- and low-resolution MS/MS spectra were used to update the database of markers for the identification of natural dyes in historical and archeological objects.

The results proved that a mild extraction method preserved quinochalcones, especially carthamin, against hydrolysis. Moreover, it was found that the composition of safflower colorants changes, depending on the dyeing conditions. Some quinochalcone *C*-glycosides (i.e., hydroxysafflor yellow A, hydroxysafflor yellow B/C, anhydrosafflor yellow B, safflor yellow A, and safflomin C) were solely found in yellow fibers, while others (including carthamin and unknown quinochalcone coded ctX) were prevalent only in red-dyed reference samples. This fact can help to determine the original color of the fabric (even if it is highly degraded, faded, or recolored) or distinguish historical technologies of dyeing with safflower.

The new MS/MS data on markers of safflower yellow and safflower red extended the analytical approach on a comprehensive identification of natural dyes in cultural heritage objects.^29,30^ The dMRM method was expanded by including the twenty-one new safflower markers, up to a total number of 188 natural compounds. Thus, safflower and its mixture with other dyes (annatto, sappanwood, orchil, and indigo) were successfully identified in the extracts of nine silk threads taken from 16th to 18th century textiles. The results have completed the picture of natural dyes present in the most valuable fabrics of European and Near Eastern origin that have been used in the vestments owned by churches from the vicinity of Krakow.

## Supporting Information Available

Chemical structures of quinochalcone colorants, proposed mechanisms of common fragmentations taking place in quinochalcone *C-*glycosides, low-resolution MS/MS spectra of anhysrosafflor yellow B isomer, anhysrosafflor yellow B, hydroxysafflor yellow A, hydroxysafflor yellow B or C, safflomin C, and ct1–ct6 compounds (*E–Z* isomers of *N*^1^*,N*^5^*,N*^10^-tri-*p*-coumaroylspermidine), and table of high-resolution MS/MS data for quinochalcone colorants (PDF)
