# Supplementary material for: Mass Spectrometry for Investigation of Natural Dyes in Historical Textiles: Unveiling the Mystery behind Safflower-Dyed Fibers
Source: J Am Soc Mass Spectrom. 2021 Sep 3;32(10):2552–66. doi: 10.1021/jasms.1c00195 (PMC8499024; doi:10.1021/jasms.1c00195)
Supplement: Supplementary file 1 — js1c00195_si_001.pdf [file js1c00195_si_001.pdf]

Mass spectrometry for investigation of natural dyes in historical textiles: unveiling the mystery behind the safflower-dyed fibers

Katarzyna Lech[1]*, Jakub Nawała[2], Stanisław Popiel[2]

[1] *Faculty of Chemistry, Warsaw University of Technology, Noakowskiego 3, 00-664 Warsaw, Poland*

[2] *Military University of Technology, Institute of Chemistry, Gen. S. Kaliskiego 2, 00-908 Warsaw, Poland*

* corresponding author: klech@ch.pw.edu.pl

Supporting Information

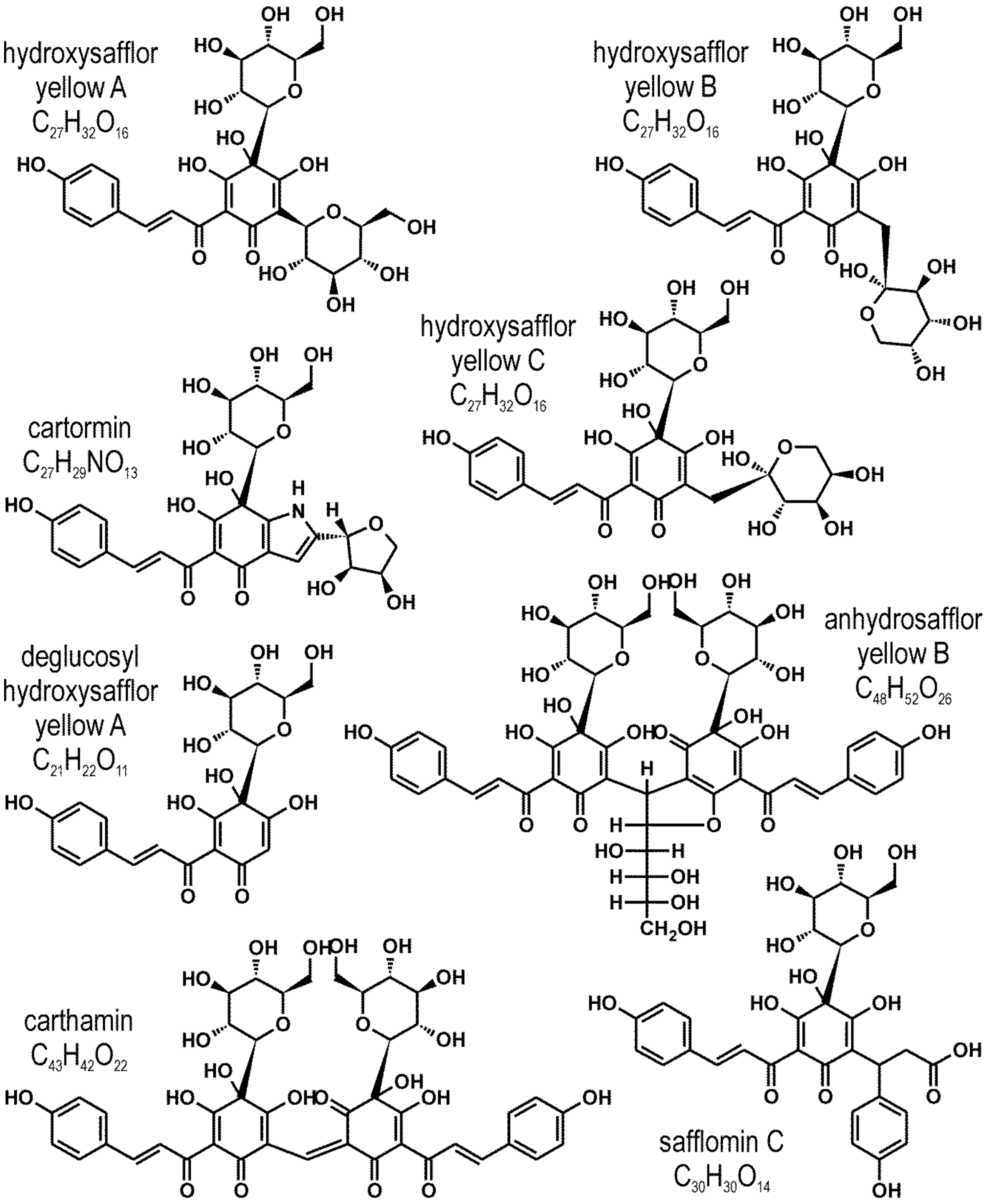

Figure S1. Chemical structures of quinochalcone colorants from safflower

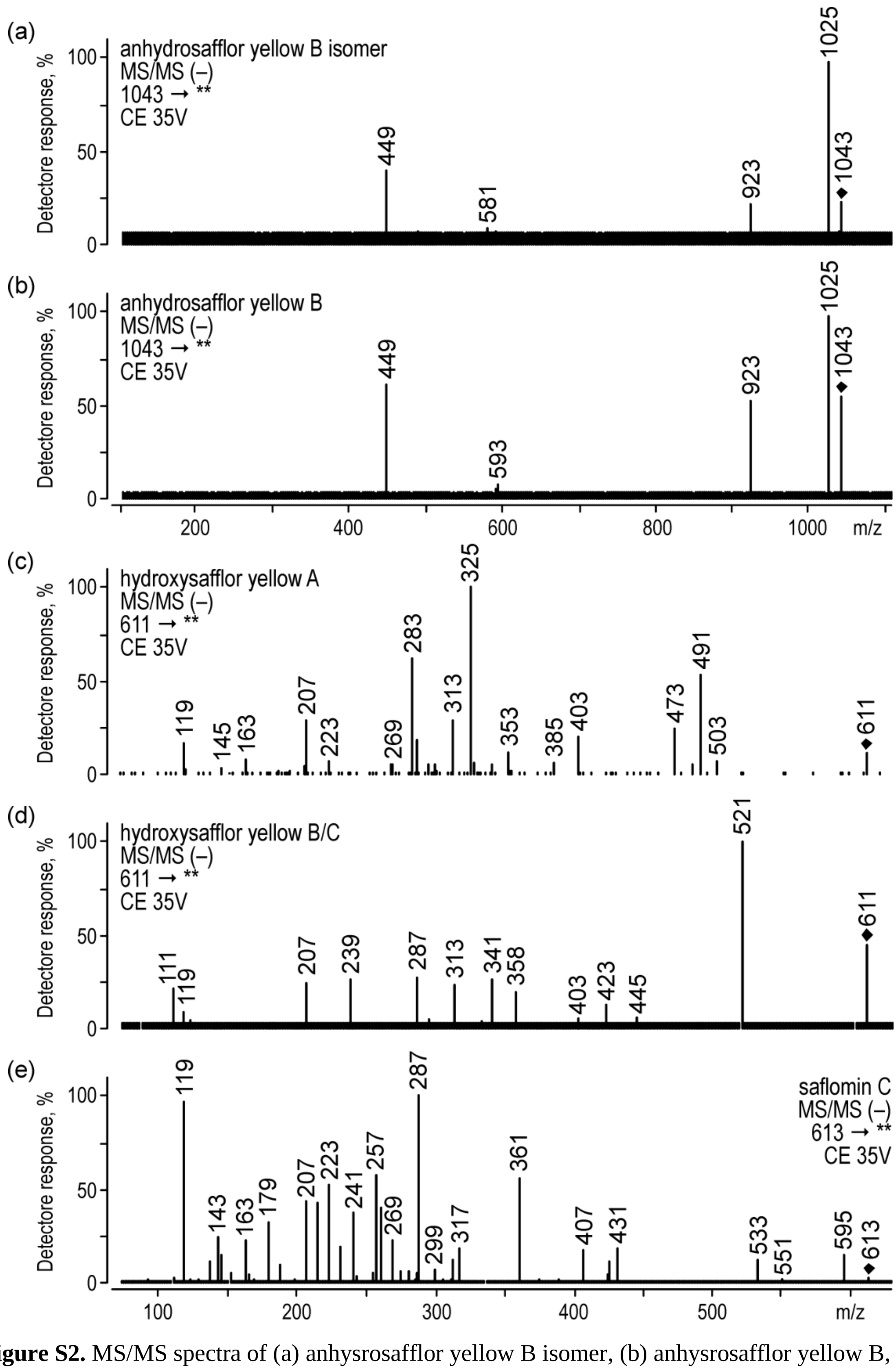

Figure S2. MS/MS spectra of (a) anhysrosafflor yellow B isomer, (b) anhysrosafflor yellow B, (c) hydroxysafflor yellow A, (d) hydroxysafflor yellow B or C, and (e) safflomin C acquired by triple quadrupole MS in negative ion mode

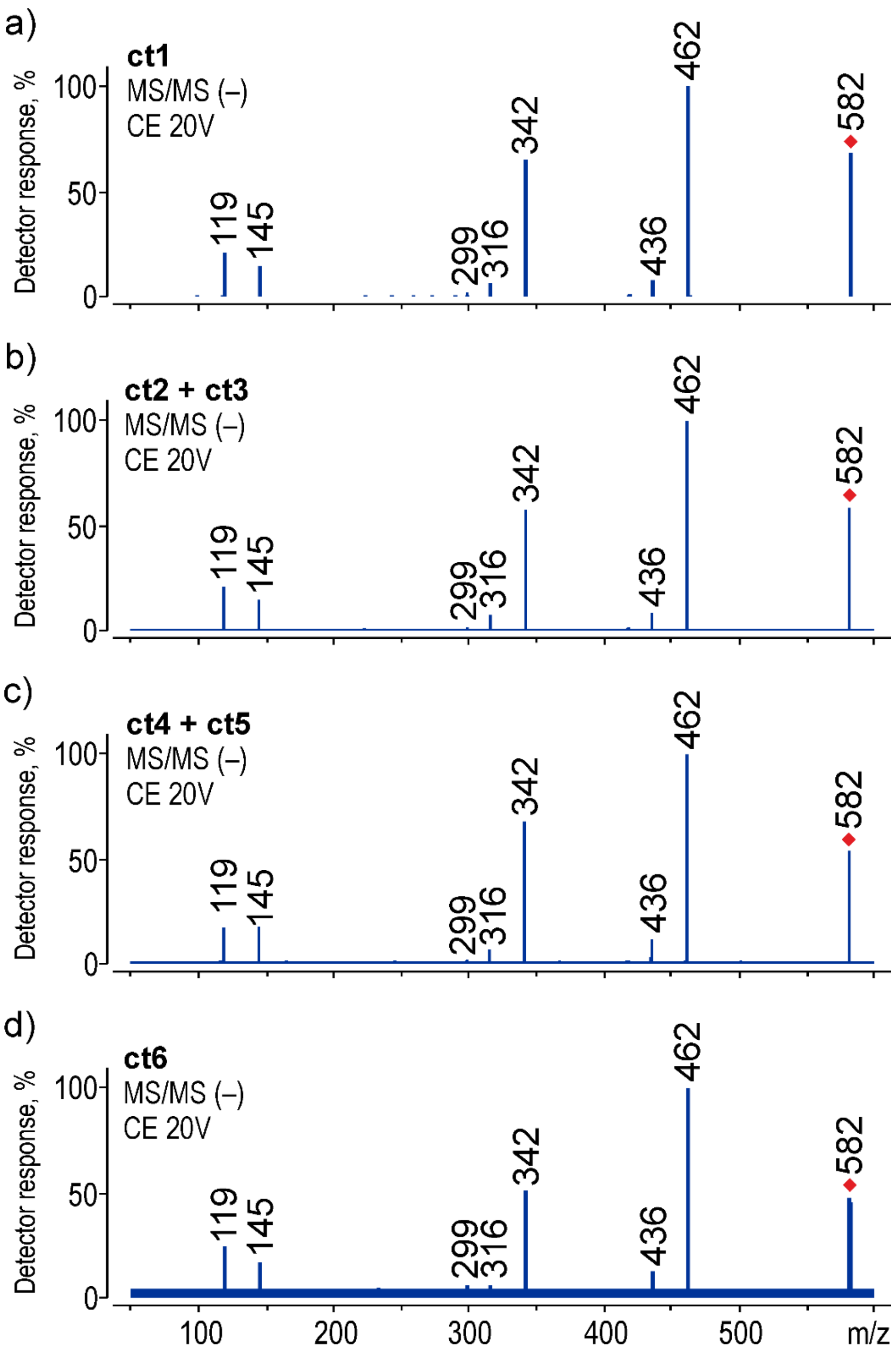

Figure S3. MS/MS spectra acquired by triple quadrupole MS in negative ion mode for ct1-ct6 compounds (*E*-*Z* isomers of N^1,N^5,N^{10}-tri-*p*-coumaroylspermidine)

Table S1. Product ions acquired using High-Resolution MS/MS in negative ion mode for quinochalcone colorants in the extract of safflower-dyed fibers

**Compound**	**[M−H]⁻, *m/z***	**Fragment ion, *m/z***	**Ion formula**	**Calculated *m/z***	**Diff., ppm**	**Abund., %**	**CE, V**
hydroxysafflor yellow A (***1***)	611.1593	→→	$C_{27}H_{31}O_{16}$	611.16066	-2.22	8.3	30
		503.11786	$C_{24}H_{23}O_{12}$	503.11840	-1.08	6.2	
		491.11777	$C_{23}H_{23}O_{12}$	491.11840	-1.29	68.0	
		473.10733	$C_{23}H_{21}O_{11}$	473.10784	-1.07	38.0	
		403.10208	$C_{20}H_{19}O_{9}$	403.10236	-0.69	28.3	
		385.09158	$C_{20}H_{17}O_{8}$	385.09179	-0.55	14.2	
		353.06537	$C_{19}H_{13}O_{7}$	353.06558	-0.60	12.3	
		328.05768	$C_{17}H_{12}O_{7}$	328.05775	-0.21	22.9	
		325.07056	$C_{18}H_{13}O_{6}$	325.07066	-0.33	100.0	
		323.05490	$C_{18}H_{11}O_{6}$	323.05501	-0.35	15.2	
		313.07062	$C_{17}H_{13}O_{6}$	313.07066	-0.15	21.6	
		295.06006	$C_{17}H_{11}O_{5}$	295.06010	-0.14	30.3	
		287.05499	$C_{15}H_{11}O_{6}$	287.05501	-0.08	15.3	
		283.06009	$C_{16}H_{11}O_{5}$	283.06010	-0.04	46.7	
		207.05020	$C_{7}H_{11}O_{7}$	207.04993	1.32	31.1	
		205.01347	$C_{10}H_{5}O_{5}$	205.01315	1.58	15.7	
		163.00298	$C_{8}H_{3}O_{4}$	163.00259	2.39	12.7	
		119.04975	$C_{8}H_{7}O$	119.04914	5.12	17.6	
hydroxysafflor yellow B/C (***3***)	611.1597	→→	$C_{27}H_{31}O_{16}$	611.16066	-1.63	96.1	30
		593.14941	$C_{27}H_{29}O_{15}$	593.15010	-1.15	15.9	
		521.12848	$C_{24}H_{25}O_{13}$	521.12897	-0.94	100.0	
		503.11804	$C_{24}H_{23}O_{12}$	503.11840	-0.72	30.0	
		473.10757	$C_{23}H_{21}O_{11}$	473.10784	-0.56	10.1	
		448.09979	$C_{21}H_{20}O_{11}$	448.10001	-0.49	40.6	
		445.11267	$C_{22}H_{21}O_{10}$	445.11292	-0.57	22.3	
		431.09711	$C_{21}H_{19}O_{10}$	431.09727	-0.39	13.2	
		423.11325	$C_{16}H_{23}O_{13}$	423.11332	-0.16	10.5	
		407.09714	$C_{19}H_{19}O_{10}$	407.09727	-0.33	17.3	
		403.10223	$C_{20}H_{19}O_{9}$	403.10236	-0.31	12.1	
		358.06827	$C_{18}H_{14}O_{8}$	358.06832	-0.14	30.8	
		353.06555	$C_{19}H_{13}O_{7}$	353.06558	-0.08	25.4	
		333.08167	$C_{13}H_{17}O_{10}$	333.08162	0.13	25.4	
		325.05551	$C_{14}H_{13}O_{9}$	325.05541	0.32	10.6	
		313.07071	$C_{17}H_{13}O_{6}$	313.07066	0.14	61.1	
		300.06296	$C_{16}H_{12}O_{6}$	300.06284	0.40	29.9	
		299.05508	$C_{16}H_{11}O_{6}$	299.05501	0.23	18.8	
		295.06021	$C_{17}H_{11}O_{5}$	295.06010	0.38	10.8	
		287.05511	$C_{15}H_{11}O_{6}$	287.05501	0.35	57.9	
		286.04730	$C_{15}H_{10}O_{6}$	286.04719	0.38	26.7	
		261.06067	$C_{10}H_{13}O_{8}$	261.06049	0.67	10.1	
		257.04465	$C_{14}H_{9}O_{5}$	257.04445	0.77	19.0	
		255.06541	$C_{15}H_{11}O_{4}$	255.06519	0.90	25.1	
		243.06548	$C_{14}H_{11}O_{4}$	243.06519	1.19	14.2	
		207.05029	$C_{7}H_{11}O_{7}$	207.04993	1.76	41.9	
		193.01357	$C_{9}H_{5}O_{5}$	193.01315	2.15	15.5	

Compound	[M−H]⁻, *m/z*	Fragment ion, *m/z*	Ion formula	Calculated *m/z*	Diff., ppm	Abund., %	CE, V
		183.02922	$C_8H_7O_5$	183.02880	2.30	11.5	
		167.03432	$C_8H_7O_4$	167.03389	2.59	12.1	
		145.02898	$C_9H_5O_2$	145.02841	3.93	13.5	
		131.03450	$C_5H_7O_4$	131.03389	4.69	21.1	
		119.04980	C_8H_7O	119.04914	5.54	21.1	
anhydrosafflor yellow B isomer (***5***)	1043.2639	→→	$C_{48}H_{51}O_{26}$	1043.26631	-2.29	100.0	20
		1025.25293	$C_{48}H_{49}O_{25}$	1025.25574	-2.74	29.1	
		923.22131	$C_{44}H_{43}O_{22}$	923.22405	-2.96	12.0	
		593.14905	$C_{27}H_{29}O_{15}$	593.15010	-1.77	2.4	
		449.10733	$C_{21}H_{21}O_{11}$	449.10784	-1.13	17.7	
anhydrosafflor yellow B (***8***)	1043.2628	→→	$C_{48}H_{51}O_{26}$	1043.26631	-3.35	19.1	20
		1025.25269	$C_{48}H_{49}O_{25}$	1025.25574	-2.98	3.2	
		923.22015	$C_{44}H_{43}O_{22}$	923.22405	-4.22	2.4	
		593.14899	$C_{27}H_{29}O_{15}$	593.15010	-1.87	7.9	
		449.10724	$C_{21}H_{21}O_{11}$	449.10784	-1.33	100.0	
deglucosyl hydroxysafflor yellow A (***11***)	449.10936	→→	$C_{21}H_{21}O_{11}$	449.10894	0.95	21.3	30
		431.09862	$C_{21}H_{19}O_{10}$	431.09837	0.58	10.4	
		311.05629	$C_{17}H_{11}O_6$	311.05611	0.57	9.3	
		299.05615	$C_{16}H_{11}O_6$	299.05611	0.13	43.1	
		298.04831	$C_{16}H_{10}O_6$	298.04829	0.08	28.8	
		297.07693	$C_{17}H_{13}O_5$	297.07685	0.28	6.7	
		287.05621	$C_{15}H_{11}O_6$	287.05611	0.34	65.8	
		286.04837	$C_{15}H_{10}O_6$	286.04829	0.29	100.0	
		285.06177	$C_{12}H_{13}O_8$	285.06159	0.63	6.8	
		281.04556	$C_{16}H_9O_5$	281.04555	0.05	17.0	
		269.04559	$C_{15}H_9O_5$	269.04555	0.16	8.7	
		261.06158	$C_{10}H_{13}O_8$	261.06159	0.04	21.2	
		259.06119	$C_{14}H_{11}O_5$	259.06120	0.03	17.5	
		257.04550	$C_{14}H_9O_5$	257.04555	0.18	13.4	
		255.06621	$C_{15}H_{11}O_4$	255.06628	0.28	17.2	
		243.06612	$C_{14}H_{11}O_4$	243.06628	0.67	16.9	
		241.05043	$C_{14}H_9O_4$	241.05063	0.84	28.4	
		207.05049	$C_7H_{11}O_7$	207.05103	2.59	43.9	
		187.03934	$C_{11}H_7O_3$	187.04007	3.89	8.6	
		178.99780	$C_8H_3O_5$	178.99860	4.45	27.2	
		153.01834	$C_7H_5O_4$	153.01933	6.48	49.3	
		119.04903	C_8H_7O	119.05024	10.15	69.1	
		111.00751	$C_5H_3O_3$	111.00877	11.33	23.6	
		97.02818	$C_5H_5O_2$	97.02950	13.64	22.5	
unknown compound X, ctX (***12***)	476.1183	→→	$C_{22}H_{22}O_{11}N$	476.11874	-0.95	79.0	20
		458.10770	$C_{22}H_{20}O_{10}N$	458.10817	-0.97	40.2	
		356.06110	$C_{14}H_{14}O_{10}N$	356.06122	-0.44	100.0	
		338.06570	$C_{18}H_{12}O_6N$	338.06591	-0.62	11.3	
		338.05057	$C_{14}H_{12}O_9N$	338.05066	-0.27	22.8	
		330.08194	$C_{13}H_{16}O_9N$	330.08196	-0.05	23.2	
		326.06589	$C_{17}H_{12}O_6N$	326.06591	-0.08	25.5	
		313.05804	$C_{16}H_{11}O_6N$	313.05809	-0.16	15.6	

Compound	[M−H]⁻, *m/z*	Fragment ion, *m/z*	Ion formula	Calculated *m/z*	Diff., ppm	Abund., %	CE, V
		308.05533	$C_{17}H_{10}O_5N$	308.05535	-0.07	34.0	
		288.07138	$C_{11}H_{14}O_8N$	288.07139	-0.04	59.7	
		286.07101	$C_{15}H_{12}O_5N$	286.07100	0.05	16.5	
		268.06049	$C_{15}H_{10}O_4N$	268.06043	0.19	69.6	
		207.05020	$C_7H_{11}O_7$	207.04993	1.32	56.4	
		206.00868	$C_9H_4O_5N$	206.00840	1.38	10.5	
		180.02950	$C_8H_6O_4N$	180.02913	2.01	47.9	
		168.02954	$C_7H_6O_4N$	168.02913	2.42	92.6	
		124.03990	$C_6H_6O_2N$	124.03930	4.81	11.6	
		119.04975	C_8H_7O	119.04914	5.12	43.8	
safflor yellow A (***13***)	593.1492	→→	$C_{27}H_{29}O_{15}$	593.15010	-1.56	14.1	30
		473.09213	$C_{19}H_{21}O_{14}$	473.09258	-0.95	11.6	
		447.11295	$C_{18}H_{23}O_{13}$	447.11332	-0.83	14.9	
		430.08914	$C_{21}H_{18}O_{10}$	430.08945	-0.72	77.3	
		425.08636	$C_{22}H_{17}O_9$	425.08671	-0.81	11.5	
		413.08646	$C_{21}H_{17}O_9$	413.08671	-0.61	14.3	
		405.10248	$C_{16}H_{21}O_{12}$	405.10275	-0.67	14.2	
		365.05020	$C_{16}H_{13}O_{10}$	365.05032	-0.33	12.6	
		353.06537	$C_{19}H_{13}O_7$	353.06558	-0.60	13.3	
		340.05756	$C_{18}H_{12}O_7$	340.05775	-0.56	13.8	
		335.05487	$C_{19}H_{11}O_6$	335.05501	-0.43	56.8	
		333.08151	$C_{13}H_{17}O_{10}$	333.08162	-0.33	10.1	
		327.07101	$C_{14}H_{15}O_9$	327.07106	-0.13	23.8	
		323.03973	$C_{14}H_{11}O_9$	323.03976	-0.08	15.8	
		310.03192	$C_{13}H_{10}O_9$	310.03193	-0.03	17.4	
		309.06052	$C_{14}H_{13}O_8$	309.06049	0.07	12.0	
		300.06281	$C_{16}H_{12}O_6$	300.06284	-0.10	10.9	
		299.05502	$C_{16}H_{11}O_6$	299.05501	0.03	20.6	
		297.06049	$C_{13}H_{13}O_8$	297.06049	-0.03	100.0	
		285.06061	$C_{12}H_{13}O_8$	285.06049	0.40	11.0	
		283.04483	$C_{12}H_{11}O_8$	283.04484	-0.05	13.1	
		261.06058	$C_{10}H_{13}O_8$	261.06049	0.32	18.8	
		237.03958	$C_{11}H_9O_6$	237.03936	0.91	21.3	
		219.02902	$C_{11}H_7O_5$	219.02880	1.02	17.8	
		211.06039	$C_{10}H_{11}O_5$	211.06010	1.39	10.2	
		207.02908	$C_{10}H_7O_5$	207.02880	1.37	28.4	
		205.01343	$C_{10}H_5O_5$	205.01315	1.36	13.9	
		195.02913	$C_9H_7O_5$	195.02880	1.69	16.5	
		193.04987	$C_{10}H_9O_4$	193.04954	1.71	23.9	
		191.03419	$C_{10}H_7O_4$	191.03389	1.62	11.1	
		179.03429	$C_9H_7O_4$	179.03389	2.24	10.2	
		177.01859	$C_9H_5O_4$	177.01824	1.98	10.7	
		165.01865	$C_8H_5O_4$	165.01824	2.49	14.3	
		163.03938	$C_9H_7O_3$	163.03897	2.53	19.5	
		153.01871	$C_7H_5O_4$	153.01824	3.09	14.1	
		151.03946	$C_8H_7O_3$	151.03897	3.24	18.1	
		145.02890	$C_9H_5O_2$	145.02841	3.41	13.2	

Compound	[M−H]⁻, *m/z*	Fragment ion, *m/z*	Ion formula	Calculated *m/z*	Diff., ppm	Abund., %	CE, V
		119.04977	C_8H_7O	119.04914	5.29	88.4	
cartormin (***14***)	574.1545	→→	$C_{27}H_{28}O_{13}N$	574.15552	-1.71	0.8	30
		466.11313	$C_{24}H_{20}O_9N$	466.11326	-0.28	4.4	
		424.10260	$C_{22}H_{18}O_8N$	424.10269	-0.22	24.2	
		411.09479	$C_{21}H_{17}O_8N$	411.09487	-0.19	16.0	
		406.09204	$C_{22}H_{16}O_7N$	406.09213	-0.21	9.9	
		378.09717	$C_{21}H_{16}O_6N$	378.09721	-0.12	10.7	
		364.08151	$C_{20}H_{14}O_6N$	364.08156	-0.14	100.0	
		364.06708	$C_{16}H_{14}O_9N$	364.06631	2.12	2.4	
		351.07379	$C_{19}H_{13}O_6N$	351.07374	0.14	8.9	
		350.06589	$C_{19}H_{12}O_6N$	350.06591	-0.07	5.6	
		346.07059	$C_{20}H_{12}O_5N$	346.07100	-1.19	1.3	
		346.05579	$C_{16}H_{12}O_8N$	346.05574	0.13	3.6	
		339.07379	$C_{18}H_{13}O_6N$	339.07374	0.15	5.5	
		338.06592	$C_{18}H_{12}O_6N$	338.06591	0.01	43.6	
		336.08670	$C_{19}H_{14}O_5N$	336.08665	0.15	9.1	
		334.07108	$C_{19}H_{12}O_5N$	334.07100	0.23	11.2	
		304.04532	$C_{14}H_{10}O_7N$	304.04518	0.46	11.6	
		291.03745	$C_{13}H_9O_7N$	291.03745	0.34	14.2	
		288.05038	$C_{14}H_{10}O_6N$	288.05026	0.42	6.0	
		286.03479	$C_{14}H_8O_6N$	286.03461	0.62	5.3	
		278.06607	$C_{13}H_{12}O_6N$	278.06591	0.56	5.3	
		260.05560	$C_{13}H_{10}O_5N$	260.05535	0.98	4.2	
		258.03995	$C_{13}H_8O_5N$	258.03970	0.96	15.2	
		244.02431	$C_{12}H_6O_5N$	244.02405	1.06	24.4	
		231.01654	$C_{11}H_5O_5N$	231.01622	1.39	7.1	
		228.02939	$C_{12}H_6O_4N$	228.02913	1.11	4.2	
		218.04512	$C_{11}H_8O_4N$	218.04478	1.54	7.7	
		216.02948	$C_{11}H_6O_4N$	216.02913	1.60	6.7	
		119.04980	C_8H_7O	119.04914	5.54	13.7	
safflomin C (***16***)	613.15684	→→	$C_{30}H_{29}O_{14}$	613.15628	0.91	100.0	30
		595.14674	$C_{30}H_{27}O_{13}$	595.14572	1.72	16.2	
		551.15601	$C_{29}H_{27}O_{11}$	551.15589	0.23	29.8	
		533.14568	$C_{29}H_{25}O_{10}$	533.14532	0.67	4.4	
		449.10956	$C_{21}H_{21}O_{11}$	449.10894	1.39	4.9	
		431.09858	$C_{21}H_{19}O_{10}$	431.09837	0.36	8.6	
		425.10940	$C_{19}H_{21}O_{11}$	425.10894	1.09	16.5	
		407.09853	$C_{19}H_{19}O_{10}$	407.09837	0.39	15.2	
		405.11943	$C_{20}H_{21}O_9$	405.11911	0.80	6.6	
		361.10828	$C_{22}H_{17}O_5$	361.10815	0.37	31.5	
		317.11886	$C_{21}H_{17}O_3$	317.11832	1.71	5.9	
		299.05631	$C_{16}H_{11}O_6$	299.05611	0.66	4.5	
		287.05615	$C_{15}H_{11}O_6$	287.05611	0.13	24.5	
		261.06175	$C_{10}H_{13}O_8$	261.06159	0.61	11.6	
		241.05046	$C_{14}H_9O_4$	241.05063	0.71	11.1	
		223.06094	$C_{11}H_{11}O_5$	223.06120	1.15	6.3	
		207.05042	$C_7H_{11}O_7$	207.05103	2.93	17.0	

Compound	[M−H]⁻, *m/z*	Fragment ion, *m/z*	Ion formula	Calculated *m/z*	Diff., ppm	Abund., %	CE, V
		165.01847	$C_8H_5O_4$	165.01933	5.22	4.8	
		119.04889	C_8H_7O	119.05024	11.32	4.5	
tri-*p*-coumaroylspermidine, ct1 (***18***)	582.25962	→→	$C_{34}H_{36}O_6N_3$	582.26096	2.3	55.0	30
		462.20211	$C_{26}H_{28}O_5N_3$	462.20345	2.9	100.0	
		436.22342	$C_{25}H_{30}O_4N_3$	436.22418	1.7	8.5	
		342.14530	$C_{18}H_{20}O_4N_3$	342.14593	1.8	57.4	
		316.16617	$C_{17}H_{22}O_3N_3$	316.16667	1.6	5.7	
		299.14075	$C_{17}H_{19}O_3N_2$	299.14012	2.1	0.1	
		145.02804	$C_9H_5O_2$	145.02950	10.1	19.0	
		119.04862	C_8H_7O	119.05024	13.6	23.4	
tri-*p*-coumaroylspermidines, ct2 + ct3 (***19 + 20***)	582.25937	→→	$C_{34}H_{36}O_6N_3$	582.26096	2.7	18.0	30
		462.20216	$C_{26}H_{28}O_5N_3$	462.20345	2.8	100.0	
		436.22347	$C_{25}H_{30}O_4N_3$	436.22418	1.6	8.2	
		342.14515	$C_{18}H_{20}O_4N_3$	342.14593	2.3	62.8	
		316.16606	$C_{17}H_{22}O_3N_3$	316.16667	1.9	6.5	
		299.13974	$C_{17}H_{19}O_3N_2$	299.14012	1.3	0.1	
		145.02804	$C_9H_5O_2$	145.02950	10.1	19.9	
		119.04863	C_8H_7O	119.05024	13.5	26.4	
tri-*p*-coumaroylspermidine, ct4 (***21***)	582.25958	→→	$C_{34}H_{36}O_6N_3$	582.26096	2.4	14.9	30
		462.20210	$C_{26}H_{28}O_5N_3$	462.20345	2.9	100.0	
		436.22326	$C_{25}H_{30}O_4N_3$	436.22418	2.1	8.1	
		342.14516	$C_{18}H_{20}O_4N_3$	342.14593	2.2	68.4	
		316.16613	$C_{17}H_{22}O_3N_3$	316.16667	1.7	7.0	
		299.13903	$C_{17}H_{19}O_3N_2$	299.14012	3.6	0.1	
		145.02801	$C_9H_5O_2$	145.02950	10.3	20.3	
		119.04861	C_8H_7O	119.05024	13.7	30.9	
tri-*p*-coumaroylspermidine, ct5 (***22***)	582.25950	→→	$C_{34}H_{36}O_6N_3$	582.26096	2.5	25.2	30
		462.20215	$C_{26}H_{28}O_5N_3$	462.20345	2.8	100.0	
		436.22335	$C_{25}H_{30}O_4N_3$	436.22418	1.9	9.4	
		342.14511	$C_{18}H_{20}O_4N_3$	342.14593	2.4	53.7	
		316.16606	$C_{17}H_{22}O_3N_3$	316.16667	1.9	5.3	
		299.14060	$C_{17}H_{19}O_3N_2$	299.14012	1.6	0.1	
		145.02801	$C_9H_5O_2$	145.02950	10.3	16.9	
		119.04861	C_8H_7O	119.05024	13.7	23.2	
tri-*p*-coumaroylspermidine, ct6 (***23***)	582.25934	→→	$C_{34}H_{36}O_6N_3$	582.26096	2.8	20.1	30
		462.20199	$C_{26}H_{28}O_5N_3$	462.20345	3.2	100.0	
		436.22331	$C_{25}H_{30}O_4N_3$	436.22418	2.0	7.0	
		342.14507	$C_{18}H_{20}O_4N_3$	342.14593	2.5	57.8	
		316.16564	$C_{17}H_{22}O_3N_3$	316.16667	3.3	5.1	
		299.13936	$C_{17}H_{19}O_3N_2$	299.14012	2.5	0.2	
		145.02801	$C_9H_5O_2$	145.02950	10.3	17.1	
		119.04861	C_8H_7O	119.05024	13.7	23.8	
carthamin (***25***)	909.20923	→→	$C_{43}H_{41}O_{22}$	909.20950	0.29	100.0	30
		501.10398	$C_{24}H_{21}O_{12}$	501.10385	0.26	63.9	
		407.09829	$C_{19}H_{19}O_{10}$	407.09837	0.20	30.9	
		287.05598	$C_{15}H_{11}O_6$	287.05611	0.46	65.1	
		234.06533	$C_{14}H_{11}O_4$	243.06628	3.92	4.1	

**Compound**	**[M−H]⁻, *m/z***	**Fragment ion, *m/z***	**Ion formula**	**Calculated *m/z***	**Diff., ppm**	**Abund., %**	**CE, V**
unknown compound Y, ctY (***26***)	477.1025	→→	$C_{22}H_{21}O_{12}$	477.10275	-0.58	100.0	20
		459.09192	$C_{22}H_{19}O_{11}$	459.09219	-0.59	28.8	
		339.04965	$C_{18}H_{11}O_{7}$	339.04993	-0.82	8.5	
		327.05023	$C_{17}H_{11}O_{7}$	327.04993	0.93	10.0	
		314.04208	$C_{16}H_{10}O_{7}$	314.04210	-0.06	13.1	
		309.03903	$C_{17}H_{9}O_{6}$	309.03936	-1.08	15.1	
		299.05524	$C_{16}H_{11}O_{6}$	299.05501	0.74	14.5	
		289.07468	$C_{8}H_{17}O_{11}$	289.07654	-6.44	34.9	
		287.05499	$C_{15}H_{11}O_{6}$	287.05501	-0.08	30.1	
		269.04443	$C_{15}H_{9}O_{5}$	269.04445	-0.06	19.8	
		207.05022	$C_{7}H_{11}O_{7}$	207.04993	1.39	17.9	
		169.01366	$C_{7}H_{5}O_{5}$	169.01315	3.00	13.7	
		153.01872	$C_{7}H_{5}O_{4}$	153.01824	3.19	10.5	
		119.04974	$C_{8}H_{7}O$	119.04914	5.06	54.2	
unknown compound Z, ctZ (***27***)	562.1548	→→	$C_{26}H_{28}O_{13}N$	562.15552	-1.30	3.7	20
		442.09766	$C_{18}H_{20}O_{12}N$	442.09800	-0.78	10.9	
		416.11859	$C_{17}H_{22}O_{11}N$	416.11874	-0.35	100.0	
		398.10797	$C_{17}H_{20}O_{10}N$	398.10817	-0.50	6.1	
		374.10803	$C_{15}H_{20}O_{10}N$	374.10817	-0.37	5.5	
		372.12881	$C_{13}H_{24}O_{12}$	372.12623	6.95	4.0	
		266.06601	$C_{12}H_{12}O_{6}N$	266.06591	0.36	7.4	
		261.06061	$C_{10}H_{13}O_{8}$	261.06049	0.44	5.7	
		207.05023	$C_{7}H_{11}O_{7}$	207.04993	1.46	4.8	
